# Electrical conductivity of warm dense silica from double-shock experiments

**DOI:** 10.1038/s41467-021-21046-1

**Published:** 2021-02-05

**Authors:** M. Guarguaglini, F. Soubiran, J.-A. Hernandez, A. Benuzzi-Mounaix, R. Bolis, E. Brambrink, T. Vinci, A. Ravasio

**Affiliations:** 1 LULI, CNRS, CEA, École Polytechnique - Institut Polytechnique de Paris, Palaiseau cedex, France; 2 Sorbonne Université, Faculté des Sciences et Ingénierie, Laboratoire d’utilisation des lasers intenses (LULI), CNRS, Paris, France; 3 École Normale Supérieure de Lyon, Université Lyon 1, Laboratoire de Géologie de Lyon, Lyon, France; 4 CEA DAM-DIF, Arpajon, France; 5 Centre for Earth Evolution and Dynamics, University of Oslo, Oslo, Norway

**Keywords:** Electronic properties and materials, Laser-produced plasmas

## Abstract

Understanding materials behaviour under extreme thermodynamic conditions is fundamental in many branches of science, including High-Energy-Density physics, fusion research, material and planetary science. Silica (SiO_2_) is of primary importance as a key component of rocky planets’ mantles. Dynamic compression is the most promising approach to explore molten silicates under extreme conditions. Although most experimental studies are restricted to the Hugoniot curve, a wider range of conditions must be reached to distill temperature and pressure effects. Here we present direct measurements of equation of state and two-colour reflectivity of double-shocked *α*-quartz on a large ensemble of thermodynamic conditions, which were until now unexplored. Combining experimental reflectivity data with numerical simulations we determine the electrical conductivity. The latter is almost constant with pressure while highly dependent on temperature, which is consistent with simulations results. Based on our findings, we conclude that dynamo processes are likely in Super-Earths’ mantles.

## Abstract (web summary)

Warm dense silica is a key component in rocky planets’ mantles, but reproducing the relevant conditions in experiments is challenging. Here the authors use a double-shock technique to achieve such conditions and measure the reflectivity in situ, providing insight into the conductivity and its possible impact on dynamo processes in super-Earths’ mantles.

## Introduction

With the discovery of numerous exoplanets slightly larger than the Earth yet likely to be rocky^1,2^, many questions have arisen regarding their properties and habitability. For instance, the existence of a dynamo process generating a magnetic field is still very uncertain^3^. Because an iron core would potentially be fully crystallised^4^, a dynamo process in long-lived deep magma oceans has been proposed^5^. It is however necessary to carefully characterise silicate melts in the megabar pressure regime^6^.

Recent ab initio calculations^7,8^ suggested that, under pressures and temperatures typical of hot young super-Earths’ interiors, the electrical conductivity of molten silica is high enough (~100 Ω^−1^cm^−1^) to generate a magnetic field from a self-sustaining dynamo. Further theoretical investigations have predicted that warm dense SiO_2_ electrical conductivity does not evolve monotonically as a function of the pressure in the megabar regime^9^, which is possibly related to coordination changes^7,9^. Experimental validations of these numerical predictions can be obtained using shock compression, where multi-megabar pressures and several thousand Kelvin temperatures can be routinely achieved. Notably, for SiO_2_^10^ and more complex silicates^11^, experimental results obtained along the principal Hugoniot exhibit changes in optical properties compatible with the semi-conducting to semi-metallic transition predicted by ab initio calculations.

Reflectivity measurements have been used to estimate the electrical conductivity, mainly via Drude or Drude–Sommerfeld models^10^. While extremely valuable for a restricted ensemble of materials^12,13^, this approach might not be generally relevant, in particular for silicates^14^. The thermodynamic conditions achieved along the Hugoniot are also often too hot with respect to planetary interiors. Moreover, temperature and pressure both increase upon single-shock compression, preventing from distinguishing their specific effects on the conductivity.

Here we present experimental data on *α*-quartz samples compressed by a double-shock to a wide ensemble of thermodynamic conditions up to now unexplored. We achieved temperatures much lower than the principal Hugoniot. Our experimental configuration allows direct measurements of the density–pressure–temperature state and shock-front reflectivity, providing a consistent physical characterisation of SiO_2_ in a yet unexplored range of conditions. With the support of ab initio simulations we were able to estimate consistently the direct-current (DC) conductivity of quartz in the megabar regime at varying temperatures. We observe that conductivity is almost independent on the pressure along isotherms. We also confirm that a dynamo process is very possible in a long-lived SiO_2_-rich magma ocean.

## Results

### Experimental campaign

**Fig. 1:**
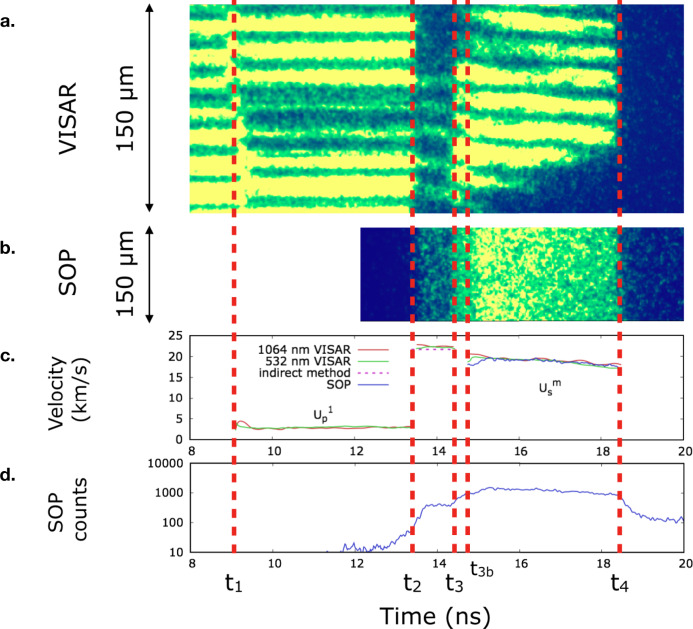
Example of an experimental output for shot A-49. Timings *t*_1_, *t*_2_, *t*_3_, *t*_3*b*_ and *t*_4_ represent the arrival time of the first shock into the sample, the arrival of the second shock into the sample, the catch-up time of the first shock by the second one, the catch-up time of the elastic precursor by the merged shock and the exit time from the sample of the merged shock. **a** Output image example of the 1064 nm VISAR; yellow (blue) indicates high (low) reflectivity in arbitrary units. **b** Output image example of the SOP; yellow (blue) indicates high (low) emission arbitrary units. **c** Time profile of the measured material velocity ${U}_{\,\text{p}\,}^{1}$ after the first shock (*t*_1_ - *t*_2_), of the second shock velocity (*t*_2_ - *t*_3_), and of the merged shock velocity ${U}_{\,\text{s}}^{\text{m}\,}$ (*t*_3*b*_ - *t*_4_). ${U}_{\,\text{s}}^{\text{m}\,}$ is not shown between *t*_3_ and *t*_3*b*_ since this time interval is too short for its precise determination from the VISAR signal. However, this measurement is not necessary for the determination of the thermodynamic state loaded by the merged shock. **d** Time profile of the measured SOP counts.

We performed three experimental campaigns on the LULI2000 laser facility (École Polytechnique, France). The technical details are available in the Methods section and in the Supplementary Notes 3. The target consisted in a CH (10 μm)/Al (70 μm)/SiO_2_ (110 or 210 μm) multi-layer stalk. The main diagnostics were a Velocity Interferometer Systems for Any Reflector (VISAR) operating at two wavelengths (532 nm and 1064 nm) and a streaked optical pyrometer (SOP). A VISAR-independent time-resolved reflectivity measurement at 532 nm was also implemented in the setup to corroborate the reflectivity measurements obtained from the VISAR fringe system. A typical diagnostic output is shown in Fig. 1. At time *t* = *t*_1_ the first weak shock breaks into the sample. The VISAR provide the Al/SiO_2_ interface material velocity considering the linear dependence in density of the quartz refractive index^15^ (see the Supplementary Notes 2). The material velocity is measured to be constant in time, attesting shock steadiness. The thermodynamic state of the quartz sample after this first compression was then directly inferred since the *α*-quartz principal Hugoniot is very well characterised (see the Supplementary Notes 1 and Supplementary Fig. 1). For each shot, the pressure, density and temperature obtained in the first shock are reported in Supplementary Table III. At time *t* = *t*_2_ the second, stronger shock breaks into the sample, causing a change of the optical properties and a rise in temperature, detected on the SOP (see Fig. 1). At *t* = *t*_3_, the two shocks merge together in a single shock and the loaded state is brought back on the principal Hugoniot, at much higher temperatures, as clearly seen in the SOP. Note that in Fig. 1 at time *t*_3*b*_ the merged shock catches up the elastic precursor, as discussed in our previous work^15^. Finally, at *t* = *t*_4_, the merged shock breaks out from the sample.

**Fig. 2:**
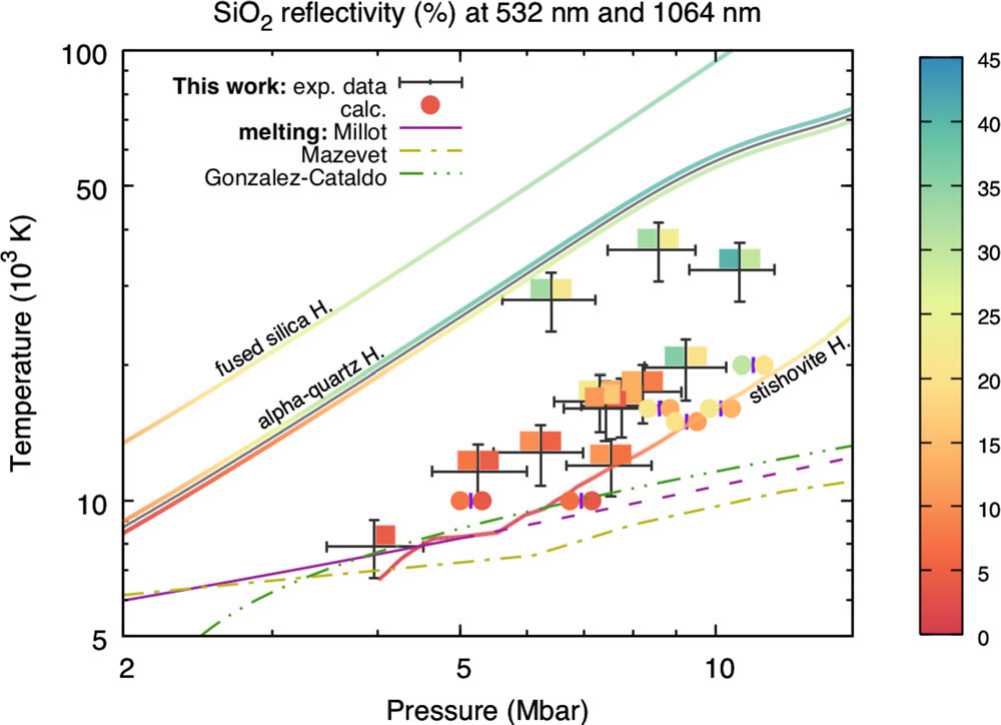
SiO_2_ reflectivity in the megabar regime. The grey crosses show the error bars on our experimental data as one standard deviation of the distribution obtained via a Monte-Carlo method (see the Supplementary Notes 6 for details). Experimentally measured second-shock-front reflectivity of silica are shown at 1064 nm (coloured squares on the left of the grey crosses) and 532 nm (on the right) as a function of temperature and pressure. The reflectivity at 1064 nm and 532 nm (left and right circles) obtained from our calculations are also shown for comparison. Reflectivity data along the fused silica Hugoniot are shown at 532 nm^16^ (SESAME 7387 has been used for the shock-velocity-to-pressure relationship). The grey solid line is the *α*-quartz Hugoniot curve^10^. Colours of the curves along the *α*-quartz Hugoniot represent the reflectivity values at 532 nm^10^ (top) and 1064 nm^30^ (bottom). Previous shock-velocity-to-pressure^31^ and shock-velocity-to-temperature^10^ relationships have been used. The stishovite Hugoniot curve has been calculated using previous data^10^, including reflectivity data at 532 nm. Different SiO_2_ melting curve predictions^9,10,32^ are also presented as solid-dashed purple, dash-dotted yellow and dash-double-dotted green lines.

The density and pressure of the double-shocked state (*ρ*_2_ and *P*_2_, respectively) have been obtained via a self-impedance mismatch analysis using the second shock velocity, itself deduced from VISAR data (details are provided in the Methods). The ensemble of the thermodynamic states reached in the double shocked sample is shown in Fig. 2 and summed up in Table 1. Pressures from 4 to 11 Mbar and temperatures from 8 × 10^3^ K to 36 × 10^3^ K have been reached, in the melt regime between the *α*-quartz and the stishovite principal Hugoniot curves. At a given pressure, temperatures up to 55% lower than along the *α*-quartz principal Hugoniot have been found for the double shocked states.

**Table 1:** Experimental (A, B, C) and simulated (S) equation-of-state data relative to the double-shocked state and second-shock-front reflectivity data at 532 nm (2*ω*_L_) and 1064 nm (*ω*_L_).

Shot/simulation	Density (g ⋅ cm^−3^)	Pressure (Mbar)	Temperature (10^3^ K)	Reflectivity 532 nm (%)	Reflectivity 1064 nm (%)	DC conductivity (10^5^ S ⋅ m^−1^)
A-15	$6.3{4}_{-0.15}^{+0.64}$	$5.2{5}_{-0.62}^{+0.74}$	11.6 ± 2.3	4.4	7.3	$0.6{4}_{-0.18}^{+0.10}$
A-40	$7.{4}_{-0.4}^{+1.6}$	$7.7{5}_{-0.81}^{+0.88}$	16.2 ± 3.2	12.9	16.3	$1.5{6}_{-0.28}^{+0.30}$
A-49	$8.{9}_{-1.4}^{+4.7}$	$6.4{0}_{-0.79}^{+0.82}$	27.9 ± 5.6	22.0	32.7	$3.0{5}_{-0.67}^{+0.96}$
A-51	$7.{6}_{-0.3}^{+2.8}$	$10.{7}_{-1.4}^{+1.1}$	32.5 ± 6.5	29.5	40.3	$5.{6}_{-1.4}^{+2.3}$
A-53	$7.{6}_{-0.4}^{+5.1}$	$8.{6}_{-1.1}^{+0.9}$	36.0 ± 7.2	23.2	32.8	$3.1{4}_{-0.68}^{+0.96}$
B-93	$7.{3}_{-0.3}^{+1.5}$	$8.2{1}_{-0.96}^{+0.90}$	17.4 ± 3.5	8.0	13.1	$1.0{2}_{-0.27}^{+0.21}$
B-98	$6.4{3}_{-0.31}^{+0.54}$	$3.9{6}_{-0.48}^{+0.56}$	7.9 ± 1.6	4.2	n.a.	n.a.
B-103	$6.8{0}_{-0.26}^{+0.96}$	$6.2{2}_{-0.74}^{+0.77}$	12.8 ± 2.6	5.0	9.7	$0.8{2}_{-0.22}^{+0.13}$
B-109	$7.{7}_{-0.4}^{+1.8}$	$9.{2}_{-1.0}^{+1.1}$	19.7 ± 4.0	19.7	35.9	$3.{5}_{-0.9}^{+1.4}$
B-111	$7.{0}_{-0.3}^{+1.1}$	$7.3{1}_{-0.85}^{+0.88}$	16.6 ± 3.3	12.6	22.4	$1.9{3}_{-0.35}^{+0.42}$
D-54	$7.2{4}_{-0.32}^{+0.97}$	$7.5{3}_{-0.86}^{+0.88}$	12.0 ± 1.7	6.9	10.4	$0.9{5}_{-0.14}^{+0.13}$
S-1	7.15	5.15	10	3.7	7.3	0.6346
S-2	7.96	6.95	10	3.6	6.5	0.4962
S-3	8.48	9.24	15	11.6	19.7	1.629
S-4	8.20	8.58	16	13.1	21.5	1.916
S-5	8.73	10.15	16	13.7	21.9	1.813
S-6	8.84	11.09	20	19.7	29.9	2.969

Experimental reflectivity data are affected by an estimated relative uncertainty of 20%. Reflectivity data for the simulated states have been obtained by applying the Fresnel formula assuming a single-shock propagation through pristine stishovite (*n*_0_ = 1.799), for states lying along, or very close to, the stishovite Hugoniot curve (S-2, S-3, S-5 and S-6) or a double-shock propagation, for off-Hugoniot states (*n*_1_ = 1.91 for S-1 and *n*_1_ = 1.89 for S-4).

### Optical properties and direct-current conductivity

A further advantage of this setup is the possibility of directly characterising the reflectivity of the double-shocked silica from VISAR measurements. Fig. 2 shows the results at 532 nm and 1064 nm (corresponding to excitation energies of 2.331 eV and 1.165 eV and denoted by the corresponding angular frequencies as 2*ω*_L_ and *ω*_L_, respectively). Our reflectivity measurements span from 3.8% at 8 × 10^3^ K, close to the melting curve, to 29% at 32 × 10^3^ K, where silica is predicted to become a dissociated fluid^16^. Reflectivity rapidly increases with temperature, approaching values previously measured on the principal Hugoniot curves of *α*-quartz, while it is weakly dependent on pressure.

A direct estimation of the optical conductivity – let alone of the DC conductivity – from the sole reflectivity measurements is generally prevented because the refractive index is a complex and frequency-dependent property. This is the reason why most of the time Drude-like models providing the conductivity frequency-dependency are used.

**Fig. 3:**
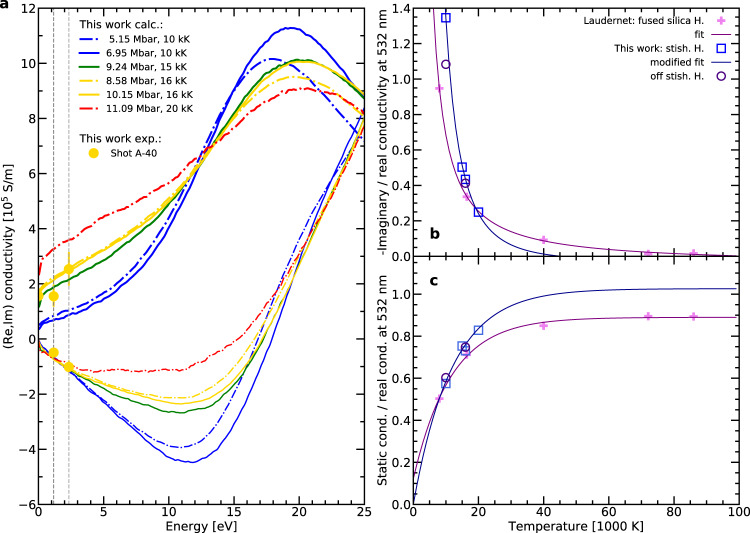
Electrical conductivity of SiO_2_ from numerical simulations. **a** Real (thick curves) and imaginary (thin curves) parts of the electrical conductivity of silica as a function of the excitation energy according to the numerical simulations. The solid curves are related to stishovite Hugoniot states; the dashed-dotted curves are related to stishovite off-Hugoniot states. The dark and light grey vertical lines identify the excitation energies corresponding to the probe-laser frequencies *ω*_L_ and 2*ω*_L_, respectively. The yellow dots represent the real and imaginary conductivity values as deduced from the reflectivity measurements at the two probe energies for shot A40 at a temperature of (16.2 ± 3.2) × 10^3^ K (detailed values are reported in Supplementary Table II). **b** Ratio between the static conductivity and the real part of the conductivity at 532 nm ($\sigma (0)/\Re [\tilde{\sigma }(2{\omega }_{\text{L}})]$) according to calculations from Laudernet et al.^17^ and this work, as a function of temperature. **c** Opposite of the ratio between the imaginary and real parts of the conductivity at 532 nm ($-\Im [\tilde{\sigma }(2{\omega }_{\text{L}})]/\Re [\tilde{\sigma }(2{\omega }_{\text{L}})]$) according to calculations from Laudernet et al.^17^ and this work, as a function of temperature. The fit curves are defined in the Methods section and in the Supplementary Notes 5.

To address this point and to better understand the behaviour of the optical properties, we complemented our experimental results with numerical ab initio simulations. Using linear response theory on Kohn–Sham Density Functional Theory (DFT) calculations, we computed the reflectivity at 532 nm and 1064 nm as in the experiments. The comparison on Fig. 2 and in Table 1 exhibits an excellent agreement between the experimental and numerical results, for both on- and off- Hugoniot states. This consistency is extremely valuable because it is the best way to benchmark the simulations results. Conductivity as a function of the excitation energy is a key information that can be extracted from the numerical simulations. It is clear from Fig. 3 that the imaginary part of the conductivity is a significant contribution to the optical properties and cannot be omitted. The numerical simulations also show that the real part of the conductivity has a non-monotonic evolution with the excitation energy. It increases with the excitation energy from the DC regime to a maximum between 17 eV and 20 eV. Our results indicate that SiO_2_ does not follow a Drude-like model, but it rather obeys to a semi-metal behaviour^8^, as already observed in the literature^17,18^.

Following these findings, in this work we adopt an alternative approach based on few key considerations derived from ab initio calculations that allow us to compute an experimentally derived DC conductivity, beyond simple Drude-like models.

As a first observation, we note that up to ~3 eV (encompassing the visible and near-IR range of our probe beams) the imaginary part of the conductivity is almost independent on both temperature and pressure (see Fig. 3a). The real part, as for it, is almost independent on the pressure, but it depends on the temperature. From a physical point of view, this difference in behaviour between real and imaginary parts suggests that the free electron density increases with temperature, while the density of scattering centers does not. As a direct consequence of these dependencies, in the 1–3 eV range, the ratio of the real to the imaginary part of the conductivity is nearly independent on the pressure and mostly dependent on the temperature. This ratio can be easily modelled by a degree 2 rational function of the temperature (see Fig. 3b and the Supplementary Notes 5 for the details of the fit). Using this fit, for both probe-laser energies we extracted the real part of the conductivity from the complex refractive index estimation. More details on the complete procedure are given in the Methods section and in the Supplementary Notes 5.

Secondly, it is worth remarking that in the same 1−3 eV energy range the derivative of the real part of the conductivity with respect to the excitation energy is nearly independent on the temperature and on the pressure (see Fig. 3a). We stress here that this observation also holds for other DFT exchange-correlation functionals since the slope is usually unchanged when using HSE06 instead of PBE^7,8^. Therefore the ratio of the DC to the 532 nm conductivity must obey a very simple dependency in temperature. Figure 3c clearly shows this behaviour, which can be modelled by a saturating exponential function of the temperature. We thus determined the DC conductivity directly from a fit on the 532 nm and 1064 nm data, as explained in the Methods section and in greater details in the Supplementary Notes 5.

**Fig. 4:**
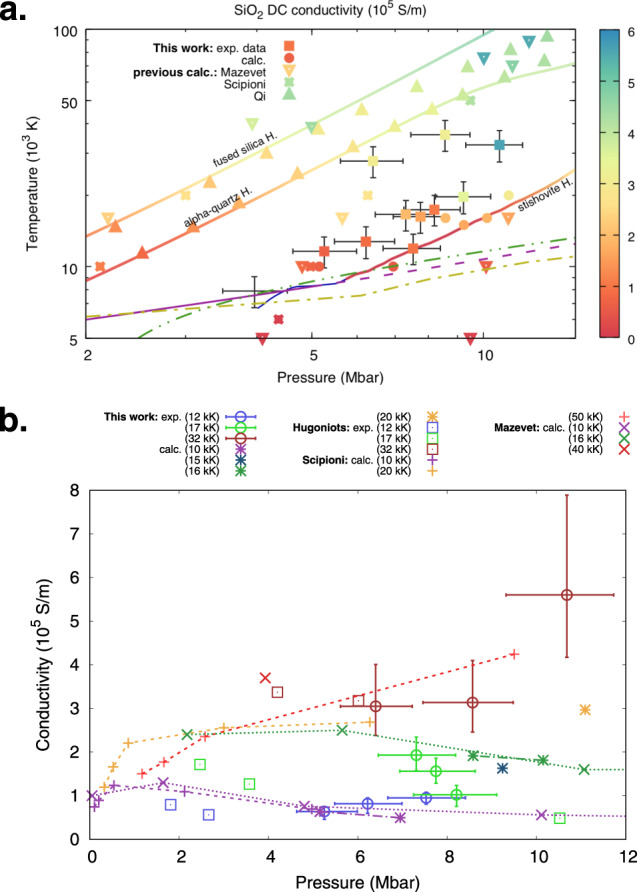
Direct current electrical conductivity of SiO_2_ in the Mbar regime. **a** DC electrical conductivity of silica (colour scale) as a function of temperature and pressure. Coloured squares are our experimental double-shock data. The grey crosses show the error bars on our data as one standard deviation of the distribution obtained via a Monte-Carlo method (see the Supplementary Notes 6 for details). Sparse data are calculations from Mazevet et al.^9^ (reversed triangles) and Scipioni et al.^7^ (carved squares). The *T*–*P* relation along the fused silica and stishovite Hugoniot curves are from Mazevet et al.^9^ and Millot et al.^10^, respectively. The static conductivity along the fused silica and *α*-quartz Hugoniot has been estimated by applying our method to reflectivity and equation-of-state data in the literature^10,16^. Melting lines as in Fig. 2. **b** SiO_2_ DC electrical conductivity as a function of pressure along three different quasi-isothermal lines, extracted from the data-set presented in panel **a**. Data from Mazevet et al.^9^ and Scipioni et al.^7^ (personal communication of the pressure values) are also shown, with thick and thin dashed lines, respectively, marking isothermal paths as a guide to the eye. The dashed lines follow the same colour code as the data-sets.

The results obtained for the static conductivity applying this procedure to our double-shock data are presented in Fig. 4a and in Table 1, along with the predictions from the numerical simulations. Conductivities of few 10^5^ S ⋅ cm^−1^ are found for our conditions, between the fused silica and stishovite principal Hugoniots. A comparison between on- and off- Hugoniot values suggests that temperature is the main driver for a conductivity increase.

## Discussion

The wide set of thermodynamic conditions reached with double-shock compression allows pushing this analysis even further and studying the pressure dependence of the DC conductivity along quasi-isothermal lines. Three isotherms – at 12 × 10^3^ K, 17 × 10^3^ K and 32 × 10^3^ K – have been extracted from our data and they are plotted in Fig. 4b. Our results at 12 × 10^3^ K show an almost constant conductivity as pressure varies, in agreement with data from Scipioni et al.^7^ and Mazevet et al.^9^ at a similar temperature – 10 × 10^3^ K. At 17 × 10^3^ K our data points suggest a decrease of conductivity for increasing pressures, providing an experimental confirmation to the calculations predictions from Mazevet et al.^9^ at 16 × 10^3^ K. At even higher temperatures – 32 × 10^3^ K – our data follow a positive slope, in agreement with what was found at 50 × 10^3^ K by Scipioni et al.^7^. The conductivity non-monotonic dependence on the pressure along isothermal paths below 20 × 10^3^ K has been interpreted either as the consequence of a change in the Si–O coordination with pressure^9^ or as the result of a high-pressure charge-ordering breaking mechanism^7^. Our results only include macroscopic measurements and do not allow us to discriminate between these two interpretations. Further experiment employing diagnostics able to probe the microscopic behaviour should be performed to help addressing this aspect.

**Fig. 5:**
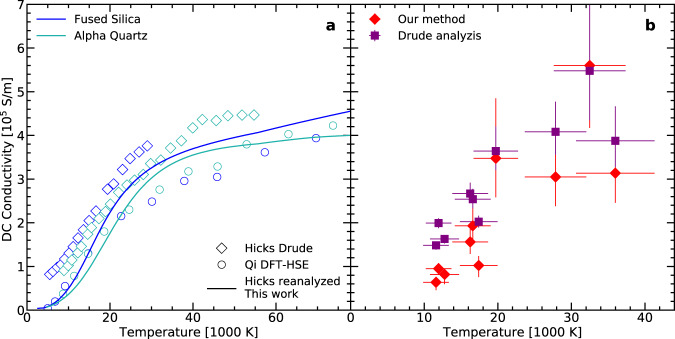
Comparison between a Drude model analysis and our method on the determination of SiO_2_ DC conductivity. **a** SiO_2_ DC electrical conductivity as a function of temperature along the fused silica (blue) and *α*-quartz (light blue) principal Hugoniot. Diamonds are conductivity values from experimental reflectivity using a Drude model^10,16^, the full lines are the results of our methodology applied to reflectivity data of Hick et al.^16^ and circles are HSE calculations^19^. **b** DC electrical conductivity values found with our methods (red diamonds) and using Drude model (violet squares) for the double shocked states presented in this work. Error bars are shown as one standard deviation of the distribution obtained via a Monte-Carlo method (see the Supplementary Notes 6 for details).

To explore the capabilities of our methodology with respect to the approaches commonly used, we have applied it to the well-established reflectivity data of fused SiO_2_ and *α*-quartz along their principal Hugoniot. The results are shown in Fig. 5a, with a comparison to the conductivity values obtained using a Drude model^10,19^ and those derived through HSE calculations^19^. At low temperatures, our approach – even if based on PBE calculations – well reproduces the conductivity values of the HSE simulations without making any strong assumptions. On the contrary, the Drude model definitively diverges from HSE, with an overestimation of the conductivity. This ascertains our method as an important improvement as HSE is assumed to provide a reliable description of the optical properties of cold silicates. At higher temperatures, the discrepancy between the Drude model, HSE calculations and our method is reduced. Full agreement is not reached for the available maximum temperature at ~60 × 10^3^ K, in agreement with calculations by Laudernet et al.^17^, showing that the conductivity along the fused silica Hugoniot does not exhibit a Drude-like behavior at least up to 86 × 10^3^ K. These observations promote our approach as a robust improvement in the estimation of SiO_2_ conductivity at extreme conditions. A similar comparison is carried out for our double-shock data set (Fig. 5b) and it leads to similar conclusions.

To sum up, we employed a double-shock compression method to generate a wide range of thermodynamic conditions. We were able to constrain the conductivity of SiO_2_ by going beyond the Drude–Sommerfeld model concurrently with state-of-the-art numerical simulations. Our results confirmed the non-trivial behavior of the conductivity as a function of the pressure predicted by numerical simulations, with important consequences for super-Earths.

Young or very close-in super-Earths as well as super-Earths in resonance can have very hot interiors^6^ with molten silicates^10^. The deepest regions could have a temperature profile going from 5000 K at 80 GPa to 9000 K at 720 GPa. By extrapolating the conductivity measurements we can estimate the conductivity of liquid SiO_2_ in the regions close to the core–mantle boundary. We estimate the conductivity at 6 × 10^4^ S ⋅ m^−1^ for SiO_2_, leading to a magnetic diffusivity *η* = 1/(*μ*_0_*σ*) of 13 m^2^ ⋅ s^−1^. Using the critical magnetic Reynolds number *Re*_m,c_ = *vl*/*η* ~ 40 for a dynamo process to occur, we can estimate that, for a convective eddy size of *l* = 100 km in a SiO_2_ magma ocean, a convective velocity *v* of 5 mm ⋅ s^−1^ in the deepest regions would be enough to sustain a dynamo process. Larger eddy sizes would only facilitate the dynamo process. Certainly, pure SiO_2_ magma oceans are unlikely as other elements such as Mg and Fe would enter the composition. Under such conditions, iron could very well stay mixed with silicates^20^ increasing even more the conductivity^21,22^. The present experimental results thus confirm the ab initio simulation predictions^7,8,22^ and support the idea that deep magma oceans are likely to produce a magnetic field in Super-Earths.

## Methods

### Experiments

We ran three experimental campaigns (A, B and D) on the LULI2000 laser facility (École Polytechnique, France). Following the technique detailed in our previous contribution^15^, we created a double-shock structure in *α*-quartz samples by focusing two drive laser pulses onto targets with the following structure: 10 μm polystyrene/70 μm aluminumc/110 or 210 μm z-cut *α*-quartz. The first pulse created a fairly weak shock that compressed the sample up to 0.2–0.6 Mbar. In this region of the Hugoniot, *α*-quartz remains transparent and is predicted to transform into the stishovite phase. The second stronger shock brought the already compressed sample at much higher pressures, up to several megabar into the liquid state. The two shocks then merged and loaded a high-pressure/high-temperature principal-Hugoniot state. Standard rear-side optical diagnostics have been used to probe the double-shocked sample: two Doppler velocity interferometers (VISAR) working at 532 nm and 1064 nm and a streaked optical pyrometer (SOP) working at different wavelength ranges in the 300–500 nm interval.

### Numerical simulations

The numerical calculations are the results of molecular dynamics (MD) simulations coupled to density functional theory (DFT) to compute the electronic densities. We used cubic simulation cells with periodic boundary conditions and containing 48 silicon atoms and 96 oxygen atoms. The temperature was controlled by a Nosé thermostat. The timestep was set at 0.5 fs for a total duration of at least 5 ps discarding the first 1 ps in the calculation of the averages. The DFT calculation was performed using the Mermin scheme^23^ with a Fermi-Dirac occupation of the Kohn–Sham orbitals. We used projector augmented wave (PAW) pseudo-potential^24^ with frozen cores: 1s^2^ for oxygen and 1s^2^2s^2^2p^6^ for silicon. To fully cover the spectrum of the occupied orbitals, we included 600 bands and solved the DFT problem in the generalised gradient approximation (GGA) with the Perdew–Burke–Ernzherhof exchange-correlation functional^25^. For these calculations, we set the plane-wave energy cut-off at 1000 eV and sampled the Brillouin zone with the Γ point only as it was sufficient for a satisfactory convergence of the pressure and energy (within 2% – see discussion in Soubiran & Militzer^8^). While for the MD we used the Vienna Ab initio Simulation Package^26^, for the calculations of the optical properties we used the code Abinit^27^. These calculations require a much higher accuracy than the MDs. We thus used snapshots extracted every 500 time steps of the trajectory and recomputed the electronic structure with more than 1000 bands to have enough unoccupied orbitals and a 4^3^ Monkhorst-Pack^28^ K-point grid to sample the Brillouin zone. From the Kohn–Sham electronic structure we obtained the real part of the conductivity via the Kubo–Greenwood formula for the conductivity. We then deduced the imaginary part of the conductivity via the Kramers–Kronig relationship. The optical properties were then computed using the complex conductivity. Details on the complete framework are given in Mazevet et al.^29^.

### Experimental determination of the optical properties and the DC conductivity

#### Optical reflectivity and complex optical conductivity

Directly from the VISAR observations, we measured the second-shock-front optical reflectivity at 532 and 1064 nm. The results are reported in Table 1. Using the Fresnel’s law we estimated the optical index behind the second shock front based on the measured reflectivity and the optical index of the single-shocked material. We have:

1 $${R}_{2}(\omega )={\left|\frac{{\tilde{n}}_{2}(\omega )-{\tilde{n}}_{1}(\omega )}{{\tilde{n}}_{2}(\omega )+{\tilde{n}}_{1}(\omega )}\right|}^{2},$$

where *R*_2_(*ω*) is the reflectivity of the second shock-front and ${\tilde{n}}_{1}(\omega )$ and ${\tilde{n}}_{2}(\omega )$ are the refractive indices of the medium loaded by the first and second shock, respectively, at the probe laser frequency *ω*. Since at low-pressure the refractive index along the *α*-quartz Hugoniot is known as a function of the density (see the Supplementary Notes 2), ${\tilde{n}}_{1}(\omega )$ was determined from the measurement of the thermodynamic state 1. We assumed the refractive index ${\tilde{n}}_{1}$ to be real.

The optical index of the double-shocked state is related to the complex conductivity by:

2 $${\tilde{n}}_{2}(\omega )={\left[1+i\frac{\tilde{\sigma }(\omega )}{{\epsilon }_{0}\omega }\right]}^{1/2},$$

where $\tilde{\sigma }(\omega )$ is the complex conductivity at *ω* and *ϵ*_0_ the vacuum permittivity.

At this point it is necessary to remember that the reflectivity represents only one measurement but we need both the real part $\Re \left[\tilde{\sigma }(\omega )\right]$ and the imaginary part $\Im \left[\tilde{\sigma }(\omega )\right]$ of the conductivity in Fresnel’s law. To circumvent this issue, we use the knowledge obtained from the ab initio simulations which allowed us to determine a relatively simple relationship between the real and imaginary parts of the conductivity:

3 $${r}^{\text{m}}(\omega ,P,T)=-\frac{\Im [\tilde{\sigma }(\omega ,T)]}{\Re [\tilde{\sigma }(\omega ,T)]}={r}^{\text{L}}(\omega ,T)\frac{{P}_{\text{st}}(T)-P}{{P}_{\text{st}}(T)-{P}_{\text{f}}(T)}+{r}^{\text{S}}(\omega ,T)\frac{P-{P}_{\text{f}}(T)}{{P}_{\text{st}}(T)-{P}_{\text{f}}(T)},$$

with *P*_st_(*T*) the pressure–temperature relationship along the stishovite Hugoniot and *P*_f_(*T*) along the fused silica one, *r*^L^ is a function resulting from the fit of the imaginary to real part ratio along the fused silica as given by the numerical simulations from Laudernet et al.^17^ and similarly along the stishovite hugoniot for *r*^S^ using our simulation results (details of the fit can be found in the Supplementary Notes 5).

Combining the previous equations we obtain a direct relationship between the reflectivity and the real part of the conductivity:

4 $${R}_{2}(\omega )={\left|\frac{{\left[1+\frac{{r}^{\text{m}}(\omega ,P,T)\Re \tilde{\sigma }(\omega )}{{\epsilon }_{0}\omega }+i\frac{\Re \tilde{\sigma }(\omega )}{{\epsilon }_{0}\omega }\right]}^{1/2}-{\tilde{n}}_{1}(\omega )}{{\left[1+\frac{{r}^{\text{m}}(\omega ,P,T)\Re \tilde{\sigma }(\omega )}{{\epsilon }_{0}\omega }+i\frac{\Re \tilde{\sigma }(\omega )}{{\epsilon }_{0}\omega }\right]}^{1/2}+{\tilde{n}}_{1}(\omega )}\right|}^{2}.$$

By measuring the density after the first shock, we can infer the optical index ${\tilde{n}}_{1}(\omega )$ (see the Supplementary Notes 2 for more details). Measuring *P* and *T* after the second shock front, we can determine *r*^m^(*ω*, *P*, *T*) as based on the numerical simulations. Finally, with the measurement of the reflectivity we self-consistently obtain the real part of the conductivity at *ω*.

The complete procedure is detailed in the Supplementary Note 5. The results for the optical conductivities at 2.331 eV (532 nm) and 1.165 eV (1064 nm) are shown in Supplementary Fig. 12.

#### From the optical to the DC conductivity

The static conductivity has been estimated from an extrapolation of the optical conductivities. In this final step we have used the ratio of the DC over the optical conductivity as a function of the pressure and temperature that we extracted from the numerical simulations (see the details in the Supplementary Note 5). The latter allowed us to establish that:

5 $${s}^{\text{m}}(\omega ,P,T)=\frac{\sigma (0,P,T)}{\Re [\tilde{\sigma }(\omega ,P,T)]}={s}^{\text{L}}(\omega ,T)\frac{{P}_{\text{st}}(T)-P}{{P}_{\text{st}}(T)-{P}_{\text{f}}(T)}+{s}^{\text{S}}(\omega ,T)\frac{P-{P}_{\text{f}}(T)}{{P}_{\text{st}}(T)-{P}_{\text{f}}(T)},$$

where the *s*^L^ and *s*^S^ functions have been adjusted on the different ab initio data from Laudernet et al.^17^ and from our own simulations, respectively. The detailed expressions of these functions are in the Supplementary Note 5. Knowing this function *s*^m^ it is then straightforward to determine the static conductivity:

6 $$\sigma (0,P,T)={s}^{\text{m}}(\omega ,P,T)\Re [\tilde{\sigma }(\omega ,P,T)].$$

We stress here again that, unlike the conductivity value itself, this ratio *s*^m^ is mostly independent on the choice of the functional in the DFT calculations and does not rely on any model assumption. Thus, even if the simulations were to predict an inaccurate absolute conductivity, its estimation from the experimental results computed using this methodology should be correct.

The values we obtained for the static conductivity are plotted in Fig. 4 and provided in Table 1.

## Supplementary information

Supplementary Information

## Data Availability

The data that support the findings of this study are available from the corresponding author upon request.

## Code availability

All simulations have been performed using ABINIT and VASP codes, that are available online (VASP is a proprietary code).
