# Supplementary material for: Electrical conductivity of warm dense silica from double-shock experiments
Source: Nat Commun. 2021 Feb 5;12:840. doi: 10.1038/s41467-021-21046-1 (PMC7865001; doi:10.1038/s41467-021-21046-1)
Supplement: Supplementary file 1 — Supplementary Information [file 41467_2021_21046_MOESM1_ESM.pdf]

Supplementary Information for
"Electrical conductivity of warm dense silica from double-shock experiments"

M. Guarguaglini[1,2], F. Soubiran[3,4], J.-A. Hernandez[1,2,5], A. Benuzzi-Mounaix[1,2], R. Bolis[1,2], E. Brambrink[1,2], T. Vinci[1,2], and A. Ravasio[1,2,*]

[1] *LULI, CNRS, CEA, École Polytechnique - Institut Polytechnique de Paris, route de Saclay, 91128 Palaiseau cedex, France*

[2] *Sorbonne Université, Faculté des Sciences et Ingénierie, Laboratoire d'utilisation des lasers intenses (LULI), CNRS, Campus Pierre et Marie Curie, 4 place Jussieu, 75252 Paris cedex 05, France*

[3] *École Normale Supérieure de Lyon, Université Lyon 1, Laboratoire de Géologie de Lyon, CNRS UMR 5276, 69364 Lyon Cedex 07, France*

[4] *CEA DAM-DIF, 91297 Arpajon, France*

[5] *Centre for Earth Evolution and Dynamics, University of Oslo, Norway*

[*] *Electronic mail:* alessandra.ravasio@polytechnique.edu

(Dated: December 23, 2020)

Supplementary Notes 1. SHOCK–MATERIAL VELOCITY RELATION ALONG THE PRINCIPAL HUGONIOT OF α-QUARTZ

In order to ensure a direct optical access to the double-shocked state, the first shock leaves the loaded sample in a transparent state. Therefore, the apparent velocity measured by the VISARs $U^1_{\rm app}$ has to be linked to $U^1_{\rm p}$ through:

$$U^1_{\rm app} = n_1 U^1_{\rm p} - (n_1 - n_0) U^0_{\rm s}, \tag{1}$$

where n_1 is the shocked refractive index and $U^0_{\rm s}$ is the first-shock velocity [1]. Solving equation 1 for $U^1_{\rm p}$ requires the knowledge of the $U_{\rm s}$-$U_{\rm p}$ relation along the principal Hugoniot of α-quartz and of the behaviour of the shocked refractive index along the same curve.

In the pressure range from $0.9 - 16$ Mbar, the $U_{\rm s}$-$U_{\rm p}$ relation along the principal Hugoniot α-quartz is very well expressed by a fit by Knudson & Desjarlais (2009) [2]. For pressures lower than the validity range of the fit, we built an interval-defined linear function to match the experimental data in the literature [3–9]. When shock-compressed up to very low material velocities ($U_{\rm p} < 0.8$ km.s^{-1}), α-quartz remains in the initial phase and the $U_{\rm s} - U_{\rm p}$ relation is linear with a positive slope. At $U_{\rm p} < 0.6$ km.s^{-1}, due to the effects of the material strength, α-quartz exhibits a composite wave structure, with an elastic precursor wave and a subsequent plastic compression wave; such effects have not been considered in this fit.

Consistently with a previous analysis [10], in the interval 0.8 km.s^{-1} $< U_{\rm p} <$ 2.4 km.s^{-1} shock-compressed quartz exhibits a mixed phase, as a direct α-quartz $\rightarrow$ stishovite phase transition is taking place. In this region, the shock velocity displays a constant value, around 5.7 km.s^{-1}. For higher $U_{\rm p} > 2.4$ km.s^{-1}, the phase transition is complete and the phase of the shocked sample is stishovite. The slope of the $U_{\rm s} - U_{\rm p}$ relation is positive again. Supplementary Figure 1 shows our fit of the $U_{\rm s} - U_{\rm p}$ relation as well as the high-pressure fit performed by [2] compared to the experimental data.

Supplementary Notes 2. MEASUREMENT OF THE REFRACTIVE INDEX OF SHOCKED α-QUARTZ

In the context of double-shock experiments, solving equation 1 to extract the real material velocity after the first shock U_{p}^{1} from the apparent velocity U_{app}^{1} measured by the VISARs requires to know not only the U_{s}-U_{p} relation along the Hugoniot, but also of the behaviour of the shocked refractive index as a function of the material velocity, $n(U_{\mathrm{p}})$ [so that $n_1 = n(U_{\mathrm{p}}^{1})$]. The refractive index can also be expressed as a function of another variable, *e.g.* the density, provided that such variable can be determined from U_{p}. After these relations have been applied, equation 1 becomes an implicit equation for U_{p}^{1} and can be solved.

A. Present knowledge and our work

The α-quartz ordinary refractive indices at ambient pressure (1.5469 and 1.5341 at 532 nm and 1064 nm, respectively) are well known [11]. Very precise refractive index measurements up to 60 kbar are available [12, 13], showing a linear relation between refractive index and density. However, the action of the first shock in our double-shock compression method brings the sample to considerably higher pressures of a few 100 kbar, spanning the whole transparency range of the principal Hugoniot curve of α-quartz. At such conditions, no refractive index data were available at the beginning of this work.

In order to extend the range along the Hugoniot in which the refractive index of quartz is known, we performed an ancillary experimental campaign at the LULI2000 facility. We generated a uniform, transparent shocked state in an α-quartz sample by focusing a 10 ns long, weak ($\sim 10^{12}$ W/cm^2) laser pulse on a multi-layer target. The thermodynamic state and the actual material velocity reached in the α-quartz sample have been obtained through an impedance mismatch analysis with the aluminum layer and, for target type (b), the lithium fluoride sample. The refractive index has then been determined from the relation between the real and apparent material velocity in the α-quartz sample. The ablator / pusher combination was 10 μm CH / 40 μm Al or 2 μm Ti / 50 μm CH / 40 μm Al. On one half of the rear-side of the Al layer, we glued a 200 μm thick α-quartz window with a thin layer of formvar resin, in order to minimise the effect on the optical properties of the interface. This target type is denoted as target type (a). For some shots, a 100 μm thick

LiF window was glued (also with formvar) on the other half of the Al surface [target type (b)]. We employed two different target types with the aim of reducing systematic errors due to the use of tabulated EOS of the standard materials (Al, LiF) in the determination of the shocked state in α-quartz. Both target schemes are provided in Supplementary Figure 2. Diagnostics included two VISARs, one at 532 nm and the other at 1064 nm.

B. Data analysis

1. Target type (a): quartz + free-aluminum.

At the shock arrival at the rear-side aluminum surface, the free-surface velocity of aluminum $U_{\mathrm{fs}}(\mathrm{Al})$ and the apparent material velocity of the aluminum/α-quartz interface $U_{\mathrm{app}}(\mathrm{SiO_2})$ have been measured. The material velocity in aluminum has been estimated from the measurement of $U_{\mathrm{fs}}(\mathrm{Al})$ by approximating the adiabatic release from the shocked state to the free-surface state ($P_{\mathrm{fs}} = 0$) using the mirrored Hugoniot curve, that is, the symmetric curve with respect to the vertical line $U_{\mathrm{p}} = U_{\mathrm{p}}(\mathrm{Al})$. Such approximation, employed as no adiabatic release data are available in this region, is justified by the fact that the entropy increase along the Hugoniot at low pressures is negligible. The approximation gives $U_{\mathrm{p}}(\mathrm{Al}) = U_{\mathrm{fs}}(\mathrm{Al})/2$. From $U_{\mathrm{p}}(\mathrm{Al})$, the other variables of the Hugoniot state reached in aluminum have been then obtained using the SESAME table 3713, which has been reproduced quite precisely by experimental studies [14]. As the shock impedance of quartz is lower than in aluminum, when the shock crosses the aluminum/quartz interface an adiabatic release takes place in the aluminum layer. The mirrored aluminum Hugoniot curve in the P-U_{p} plane has been used to approximate such release in aluminum towards state reached in α-quartz. The α-quartz Hugoniot curve from our fit (see Section Supplementary Notes 1) has been used. The intersection between the aluminum release and the quartz Hugoniot gave the reached (P, U_{p}) point in shocked quartz. A scheme of the impedance mismatch analysis is provided in Supplementary Figure 3 (a). The refractive index of shocked quartz has then been extracted by inverting equation 1:

$$n = \frac{U_{\mathrm{app}} - U_{\mathrm{s}} n_0}{U_{\mathrm{p}} - U_{\mathrm{s}}}. \tag{2}$$

2. *Target type (b): quartz + lithium fluoride.*

For this type of target, we measure the apparent material velocity of the aluminum / LiF and of the aluminum / α-quartz interface, $U_{\mathrm{app}}(\mathrm{LiF})$ and $U_{\mathrm{app}}(\mathrm{SiO_2})$, respectively, at the shock arrival at the rear-side aluminum surface. The principal Hugoniot and shocked refractive index of LiF are known from SESAME table 7270 and Rigg et al. (2014) [15], respectively, and have been used to estimate the true material velocity in LiF $U_{\mathrm{p}}(\mathrm{LiF})$ and thus the reached pressure $P(\mathrm{LiF})$. The state reached in aluminum has been determined through an impedance mismatch analysis between aluminum and LiF, using the mirrored Hugoniot of Al to approximate its adiabatic release towards the conditions reached in LiF. Finally, the (U_{p}, P) state reached in quartz has been measured via an impedance mismatch analysis between aluminum and quartz. A scheme of the analysis is provided in Supplementary Figure 3 (b). The refractive index of shocked-quartz has been measured using equation 2.

C. Results

Our results (provided in Supplementary Table I) show that the α-quartz refractive index dependence on density can be reasonably assumed to be linear up to 4.2 g.cm^{-3} (320 kbar). Supplementary Figure 4 shows the experimental results of this work together with previous low-compression data from previous studies [12, 13]. The linear fits performed on a previous data set [12] and on the results of this work, both forced to reproduce the known refractive index value at initial density, exhibit surprisingly similar slopes.

Supplementary Notes 3. EXPERIMENTAL SETUP OF THE MAIN CAMPAIGNS

In our experimental campaigns, drive laser pulses at 527 nm were delivered by the North and South chains of the LULI2000 facility (École Polytechnique, France). The first pulse delivered $70 - 130$ J within a duration of 10 ns; the second one $300 - 500$ J in 1.5 or 2 ns. Phase plates guaranteed a uniform illumination of the target over a 500 μm diameter. A time profile plot of the two laser pulses for shot A-40 is provided in Supplementary Figure 5. The targets were mounted on cone-shaped plastic washers. A scheme of the target is shown in Supplementary Figure 6. Diagnostics included two VISARs, one working at 532 nm and the other at 1064 nm. They were illuminated by a probe laser whose FWHM duration was $\sim$ 20 ns at 532 nm and $\sim$ 10 ns at 1064 nm. VISAR velocity-per-fringe parameters were 6.11 and 15.94 km.s^{-1} at 532 nm and 1064 nm, respectively. A VISAR-independent reflectivity diagnostics (reflectometer) consisting in a streak camera illuminated by the probe laser at 532 nm was included in the setup to corroborate the VISAR reflectivity measurements. An example of a raw image of the VISAR at 1064 nm is included in the main text. Supplementary Figure 7 shows a further example of the VISARs at the two wavelengths. In the same figure we also show the typical region considered in the analysis. A streaked optical pyrometer (SOP) collected the thermal emission of the shocked target in a spectral range included in the $300 - 500$ nm interval.

Supplementary Notes 4. HYDRODYNAMICS OF THE DOUBLE-SHOCK COMPRESSION

In our previous article [1] we provided the details of the double shock compression technique as well of the analysis procedure used to infer the thermodynamic state. We also included schemes clarifying the propagation history of the different waves and the interfaces probed by the diagnostics at each temporal interval. To facilitate the reading of the present article, we report here similar representations for our quartz targets. In particular we include a time-space scheme (Supplementary Figure 8), a target scheme clarifying the probed interfaces (Supplementary Figure 9), and an output of a 1D simulation using the code MULTI (Supplementary Figure 10). Moreover, for each shot we resume the thermodynamic state of the target after the first compression, i.e. the starting conditions for the second shock) in Supplementary Table III.

Supplementary Notes 5. ELECTRICAL CONDUCTIVITY OF WARM DENSE SILICA

A. Estimation of the conductivities at 532 nm and 1064 nm

1. From the reflectivity to the conductivity

The reflectivity of the first shock front was negligible ($\leq 1\%$), due to the low change in the refractive index in the first compression. We could therefore assume that we essentially measured the second-shock front reflectivity $R_2(\omega)$ at the two probe laser frequencies $\omega = \omega_\mathrm{L}$, $2\omega_\mathrm{L}$ (corresponding to the wavelengths of 1064 nm and 532 nm, respectively).

$R_2(\omega)$ can be linked to the refractive indices of states 1 and 2 at the same frequency, $\tilde{n}_1(\omega)$ and $\tilde{n}_2(\omega)$, respectively, via the Fresnel's equation (equation 1 of the main article). The complex refractive index of the double-shocked state 2 can be linked to its complex electrical conductivity via equation 2 in the main article. Only the solution with a positive imaginary part ($\Im(\tilde{n}) > 0$) of the complex square root in equation 2 in the main article has to be taken, as the other would produce non-physical effects such as exponentially increasing electric fields inside the medium.

At this point, the complex optical conductivity of state 2, $\tilde{\sigma}_2(\omega)$, cannot be univocally determined without further assumptions. Indeed, the system to be solved is composed only by one equation linking the measured reflectivity to the complex optical conductivity, but has two unknowns, $\Re[\tilde{\sigma}_2(\omega)]$ and $\Im[\tilde{\sigma}_2(\omega)]$. Therefore, an additional relation including the unknowns must be considered. In the Drude model, such relation is implicitly provided by the functional form of the frequency dependence of the complex conductivity:

$$\tilde{\sigma}(\omega) = \frac{1}{1 - i\omega\tau}\sigma(0), \tag{3}$$

where τ is the electron-ion scattering time and $\sigma(0)$ is the (purely real) DC conductivity.

We wanted to find a more significative relation $\Re[\tilde{\sigma}_2(\omega)]$ and $\Im[\tilde{\sigma}_2(\omega)]$ at both probe laser frequencies ($\omega = \omega_\mathrm{L}$, $2\omega_\mathrm{L}$) without the use of the simplistic Drude approach. To do so, we have first interpolated the ratio between $\Re[\tilde{\sigma}_2(\omega)]$ and $\Im[\tilde{\sigma}_2(\omega)]$ along the fused silica and stishovite Hugoniot conditions explored by the ab initio simulations from [16] and of this work, respectively (see Supplementary Figure 11), which we have previously validated by comparing our reflectivity data to their predictions. The calculated ratios are shown in

Fig. 3b of the main text as a function of temperature. We performed a fit on both datasets with the function

$$r(\omega, T) = a(\omega) + \frac{b(\omega)}{T} + \frac{c(\omega)}{T^2}, \tag{4}$$

where

$$r(\omega, T) = -\frac{\Im[\tilde{\sigma}(\omega, T)]}{\Re[\tilde{\sigma}(\omega, T)]} \tag{5}$$

is the opposite of the ratio between the imaginary and real part of the conductivity at a given probe laser frequency.

To estimate $r(\omega)$ to our conditions, which are off both the fused silica and the stishovite Hugoniot curves, we considered their pressure and temperature dependence. In particular, the effect of pressure on $r(\omega)$ appears to be weak. Therefore, we operated a linear interpolation between the values from [16] and this work along isothermal lines:

$$r^{\mathrm{m}}(\omega, P, T) = r^{\mathrm{L}}(\omega, T)\frac{P_{\mathrm{stishovite}}(T) - P}{P_{\mathrm{stishovite}}(T) - P_{\mathrm{fused}}(T)} + r^{\mathrm{S}}(\omega, T)\frac{P - P_{\mathrm{fused}}(T)}{P_{\mathrm{stishovite}}(T) - P_{\mathrm{fused}}(T)}, \tag{6}$$

where $r^{\mathrm{m}}(\omega, P, T)$ is the estimated ratio at a certain frequency for a pressure P and a temperature T, $r^{\mathrm{L}}(\omega, T)$ and $r^{\mathrm{S}}(\omega, T)$ are the ratios given by the fit on the calculations from Laudernet et al. (2004) [16] and this work, respectively, and $P_{\mathrm{stishovite}}(T)$ and $P_{\mathrm{fused}}(T)$ are the Hugoniot pressure of stishovite and fused silica at the temperature T, respectively. This interpolation has physical meaning for $P_{\mathrm{fused}}(T) < P < P_{\mathrm{stishovite}}(T)$, which is always the case for our data.

2. *Results*

We could write the conductivity as:

$$\tilde{\sigma}_2(\omega) = \Re[\tilde{\sigma}_2(\omega)][1 - i \cdot r^{\mathrm{m}}(\omega, P_2, T_2)], \tag{7}$$

where P_2 and T_2 are the measured pressure and temperature of the double-shocked state, respectively. Equation 7 has been substituted into equation 2 of the main article and then into equation 1 of the main article to infer the real part of the conductivity at $\omega = \omega_{\mathrm{L}}$, $2\omega_{\mathrm{L}}$ from the measurement of the second-shock front reflectivity at the same frequency.

The electrical conductivity values at 532 nm and 1064 nm of the double-shocked states found in such a way are provided in Supplementary Table II. They are also shown in Supplementary Figure 12, where they are compared with the values of the simulations of this work.

B. Estimation of the DC conductivity

According to the calculations of Laudernet et al. (2004) [16] and of this work, the slope of the real part of the conductivity as a function of frequency is almost independent on temperature or pressure in the low-frequency range (around $0 - 3$ eV), which covers our probe laser frequencies $\omega_{\rm L}$ and $2\omega_{\rm L}$ (see Figure 3 in the main article). We thus determined the DC value of the conductivity $\sigma(0)$ and the real part of the values at 1.165 eV and 2.33 eV ($\Re[\tilde{\sigma}(\omega_{\rm L})]$ and $\Re[\tilde{\sigma}(2\omega_{\rm L})]$, respectively) directly from a linear fit on the data in the visible range. Our approach consists in determining the ratio between static and optical values of the conductivity from the ab initio simulations and then search for the static conductivity value that reproduces, via the aforementioned ratio, the optical values as close as possible to those we estimated in Section Supplementary Notes 5 A.

Therefore, for each state simulated in Laudernet et al. (2004) [16] or in this work, we extracted the ratio between the static conductivity and the real part of the conductivity at $\omega = \omega_{\rm L}$ and $\omega = 2\omega_{\rm L}$:

$$s(\omega, T) = \frac{\sigma(0,T)}{\Re[\tilde{\sigma}(\omega,T)]}. \tag{8}$$

In order to determine the temperature dependency of such ratio, we fitted the datasets of Laudernet et al. (2004) [16] and this work with a properly designed function

$$s(\omega, T) = s^{\rm in}(\omega) + \left[s^{\rm sat}(\omega) - s^{\rm in}(\omega)\right]\left[1 - \exp\left(-\frac{T}{T^{\rm sc}(\omega)}\right)\right]. \tag{9}$$

The free parameters of the fit are $s^{\rm in}(\omega)$, $s^{\rm sat}(\omega)$, and $T^{\rm sc}(\omega)$. From now on, the best fits on the datasets of Laudernet et al. (2004) [16] and this work will be called $s^{\rm L}(\omega,T)$ and $s^{\rm S}(\omega,T)$, respectively. The datasets and the corresponding fitting functions are provided in Figure 3 of the main article.

The functions $s^{\rm L}(\omega,T)$ and $s^{\rm S}(\omega,T)$ assume fairly close values at similar temperatures, despite being relative to much different pressure values: the pressure dependence appears to be much weaker than the temperature one. Therefore, we supposed that the pressure dependence of the ratio $s^{\rm m}(\omega,P,T)$ can be approximated by a linear interpolation of the values $s^{\rm L}(\omega,T)$ and $s^{\rm S}(\omega,T)$:

$$s^{\rm m}(\omega,P,T) = s^{\rm L}(\omega,T)\frac{P_{\rm stishovite}(T) - P}{P_{\rm stishovite}(T) - P_{\rm fused}(T)} + s^{\rm S}(\omega,T)\frac{P - P_{\rm fused}(T)}{P_{\rm stishovite}(T) - P_{\rm fused}(T)}. \tag{10}$$

At this point, the problem was to find the static conductivity that produced, via the aforementioned ratios, the closest optical conductivity values to the values obtained from

the experimental data. To do so, we considered a free parameter $\sigma^{\mathrm{m}}(0)$ representing the static conductivity and calculated the corresponding optical values $\sigma^{\mathrm{m}}(\omega_{\mathrm{L}})$ and $\sigma^{\mathrm{m}}(2\omega_{\mathrm{L}})$ for each value of that parameter via:

$$\sigma^{\mathrm{m}}(\omega) = \frac{\sigma^{\mathrm{m}}(0)}{s^{\mathrm{m}}(\omega, P, T)} \tag{11}$$

Then, we searched for the value of $\sigma^{\mathrm{m}}(0)$ that minimised a displacement function $D[\sigma^{\mathrm{m}}(0)]$, defined as the sum of the squares of the displacement between the optical conductivity predicted by the model and that estimated from the experimental data, rescaled with the uncertainty associated to the latter:

$$D[\sigma^{\mathrm{m}}(0)] = \left[\frac{\sigma^{\mathrm{m}}(\omega_{\mathrm{L}}) - \sigma(\omega_{\mathrm{L}})}{\Delta_{\pm}\sigma(\omega_{\mathrm{L}})}\right]^2 + \left[\frac{\sigma^{\mathrm{m}}(2\omega_{\mathrm{L}}) - \sigma(2\omega_{\mathrm{L}})}{\Delta_{\pm}\sigma(2\omega_{\mathrm{L}})}\right]^2. \tag{12}$$

Since the error bars on $\sigma(\omega)$ are asymmetric, the upper and lower values $\Delta_{+}\sigma(\omega)$ and $\Delta_{-}\sigma(\omega)$ have been considered if the predicted optical conductivity value $\sigma^{\mathrm{m}}(\omega)$ was respectively higher or lower than the value estimated from the experimental data $\sigma(\omega)$.

In Supplementary Figure 13 (left), we show the estimated static conductivity of the double-shocked state for a given shot, together with the optical values at ω_{L} and $2\omega_{\mathrm{L}}$ and the linear dependency on energy given by the model. We provide on the right of Supplementary Figure 13 the displacement function D against the varying static conductivity $\sigma^{\mathrm{m}}(0)$ for shot D-54.

Supplementary Notes 6. ERROR ESTIMATION

A. Measurement of the refractive index

Errors in the refractive index measurements (appearing in Supplementary Table I) depend on the apparent material velocity measure done with the VISARs and on the equation of state measure done with through impedance mismatch. A Monte-Carlo routine has been used to estimate the error on density and pressure of the reached state and on its refractive index. The data analysis code has been run 1000 times by varying the apparent material velocity in quartz and either the aluminum free-surface velocity [target type (a)] or the apparent material velocity in LiF [target type (b)]. Each input was extracted from a Gaussian distribution whose mean and standard deviation correspond to the measured value and its estimated uncertainty, respectively. The uncertainties on density, pressure, and refractive index have been estimated as the standard deviations of the output population.

B. Double-shocks: timings and velocities

Errors on timings depend on the VISARs and SOP time resolution and on the planarity of the shock profiles. In a typical case, uncertainties were about 5 pixels, or about 100 ps.

Velocities directly measured by the VISARs (U_p^1 and U_s^m) are affected by an error due to the uncertainty on the fringe shift measure. We estimated it as 1/10 of a fringe. In the case of a material velocity measure, such as U_p^1, also the low-pressure $U_\mathrm{s} - U_\mathrm{p}$ relation and the shocked refractive index are error sources. A Taylor expansion of the $U_\mathrm{s} - U_\mathrm{p}$ relation thus gives the correct error estimation. The measurement of the merged shock velocity right after the merging, $U_\mathrm{s}^\mathrm{m}(t_3)$, is also affected by the uncertainty associated to the linear fit on $U_\mathrm{s}^\mathrm{m}(t)$ to extract that value. The indirect measurement of the second-shock velocity U_s^1 [1] was affected by the uncertainties on the material velocity U_p^1 and the timings t_1, t_2, and t_3.

C. Double-shocks: thermodynamic state

Errors on density and pressure have been estimated through a Monte-Carlo routine. The analysis has been run 1000 times changing the following inputs: the thickness of the quartz sample ($1 - 2$ μm, indicated by the supplier), the timings t_1, t_2, t_3, t_{3b}, and t_4, and the

velocities U_p^1 and $U_s^\mathrm{m}(t_{3b})$. Errors on temperature have multiple sources: uncertainty on the SOP calibration parameter, on the measure of the counts on the SOP image, on the measure of reflectivity. Typical relative errors were of about 20%.

D. Double-shocks: reflectivity and conductivity

Uncertainties on the second-shock front reflectivity are mainly due to calibration. Other sources of error include the noise in the VISAR output and the spatial non-uniformity of the shock fronts. Typical relative error bars are of about 20%.

The estimation of the optical conductivity values is delicate and depends on several parameters. The most important error source for $\sigma(\omega)$ is the second-shock reflectivity measure at the same wavelength, $R_2(\omega)$. Other error sources are the double-shocked density, and the double-shocked state pressure and temperature (which influence the estimation of the ratio between imaginary and real part of the conductivity, as detailed in Section Supplementary Notes 5 A 1). The errors on electrical conductivity have been estimated using a Monte-Carlo routine. The routine repeated the analysis 5000 times varying the following inputs: ρ_2, T_2, P_2, $R_2(\omega_\mathrm{L})$, $R_2(2\omega_\mathrm{L})$. Supplementary Figure 15 shows a subset of the conductivity output data as a function of the reflectivity input.

The same Monte-Carlo routine employed for the determination of the uncertainties on the optical conductivity values has been used to calculate the error bars on $\sigma(0)$. They are reported in Supplementary Table II.

SUPPLEMENTARY TABLES AND FIGURES

Supplementary Table I. α-quartz refractive index shots. Targets of type (a) allow the simultaneous measurement of the apparent velocity in quartz and of the aluminum free-surface velocity. Targets of type (b) allow the measurement of the apparent velocity in lithium fluoride instead that of the aluminum free-surface.

Shot number	Target type	Density (g.cm^{-3})	Pressure (Mbar)	Refractive index
C-39	(b)	4.01 ± 0.08	0.292 ± 0.012	1.760 ± 0.042
C-46	(a)	4.22 ± 0.06	0.320 ± 0.008	1.810 ± 0.034
C-48	(a)	3.10 ± 0.03	0.124 ± 0.007	1.616 ± 0.022
C-51	(b)	3.47 ± 0.06	0.203 ± 0.011	1.684 ± 0.034
C-55	(a)	4.05 ± 0.06	0.298 ± 0.008	1.812 ± 0.032

Supplementary Table II. Conductivity data for double-shocked α-quartz (experiments A, B, and D) and simulations (S) on warm dense silica. ω_{L} indicates a wavelength of 1064 nm, $2\omega_{\mathrm{L}}$ a wavelength of 532 nm. The real and imaginary parts of the conductivity at each wavelength is shown together with its positive and negative error bar. Conductivity values are expressed in 10^5 S/m.

Shot/sim.	$\Re\left[\sigma(2\omega_{\mathrm{L}})\right]$	$-\Im\left[\sigma(2\omega_{\mathrm{L}})\right]$	$\Re\left[\sigma(\omega_{\mathrm{L}})\right]$	$-\Im\left[\sigma(\omega_{\mathrm{L}})\right]$
A-15	$1.16^{+0.25}_{-0.52}$	$0.97^{+0.15}_{-0.44}$	$0.81^{+0.16}_{-0.20}$	$0.49^{+0.10}_{-0.17}$
A-40	$2.54^{+0.62}_{-0.60}$	$1.02^{+0.20}_{-0.24}$	$1.55^{+0.43}_{-0.38}$	$0.49^{+0.14}_{-0.15}$
A-49	$3.5^{+1.2}_{-1.0}$	$0.45^{+0.15}_{-0.12}$	$3.3^{+1.7}_{-1.1}$	$0.40^{+0.21}_{-0.14}$
A-51	$6.1^{+2.8}_{-2.0}$	$0.46^{+0.23}_{-0.15}$	$5.7^{+4.0}_{-2.3}$	$0.47^{+0.33}_{-0.17}$
A-53	$3.5^{+1.3}_{-1.0}$	$0.24^{+0.10}_{-0.07}$	$3.1^{+1.6}_{-1.1}$	$0.24^{+0.12}_{-0.07}$
B-93	$1.38^{+0.43}_{-0.61}$	$0.47^{+0.12}_{-0.21}$	$1.16^{+0.32}_{-0.30}$	$0.32^{+0.09}_{-0.10}$
B-98	$0.88^{+0.22}_{-0.23}$	$1.88^{+0.32}_{-0.48}$	n.a.	n.a.
B-103	$1.22^{+0.30}_{-0.61}$	$0.84^{+0.16}_{-0.42}$	$1.03^{+0.22}_{-0.23}$	$0.53^{+0.11}_{-0.16}$
B-109	$3.8^{+1.2}_{-1.0}$	$0.97^{+0.26}_{-0.25}$	$4.9^{+2.9}_{-1.8}$	$1.05^{+0.61}_{-0.45}$
B-111	$2.45^{+0.61}_{-0.60}$	$0.93^{+0.18}_{-0.23}$	$2.38^{+0.80}_{-0.64}$	$0.72^{+0.24}_{-0.24}$
D-54	$1.63^{+0.29}_{-0.35}$	$1.44^{+0.18}_{-0.31}$	$1.12^{+0.23}_{-0.22}$	$0.72^{+0.14}_{-0.20}$
S-1	1.053	1.140	0.8359	0.6893
S-2	0.8627	1.161	0.6836	0.6458
S-3	2.162	1.088	1.876	0.7204
S-4	2.562	1.056	2.232	0.7030
S-5	2.482	1.077	2.141	0.7277
S-6	3.586	0.8944	3.256	0.6982

Supplementary Table III. Conditions reached after the first shock loading in double-shock shots on α-quartz targets. Temperatures without an uncertainty correspond to states lying in the Hugoniot temperature plateau from $0.12 - 0.36$ Mbar, where an α-quartz $\rightarrow$ stishovite phase transition is taking place.

Shot number	ρ_1 (g.cm^{-3})	P_1 (Mbar)	T_1 (K)
A-15	4.79 ± 0.10	0.64 ± 0.18	3050 ± 640
A-40	4.67 ± 0.04	0.46 ± 0.05	2490 ± 150
A-49	3.91 ± 0.19	0.28 ± 0.03	2190
A-51	4.73 ± 0.06	0.54 ± 0.09	2730 ± 280
A-53	3.81 ± 0.22	0.26 ± 0.03	2190
B-93	4.67 ± 0.10	0.46 ± 0.12	2490 ± 370
B-98	4.67 ± 0.03	0.46 ± 0.04	2490 ± 110
B-103	4.89 ± 0.10	0.85 ± 0.22	3840 ± 840
B-109	4.66 ± 0.04	0.45 ± 0.05	2450 ± 140
B-111	4.74 ± 0.05	0.56 ± 0.07	2780 ± 240
D-54	4.73 ± 0.02	0.54 ± 0.03	2730 ± 90

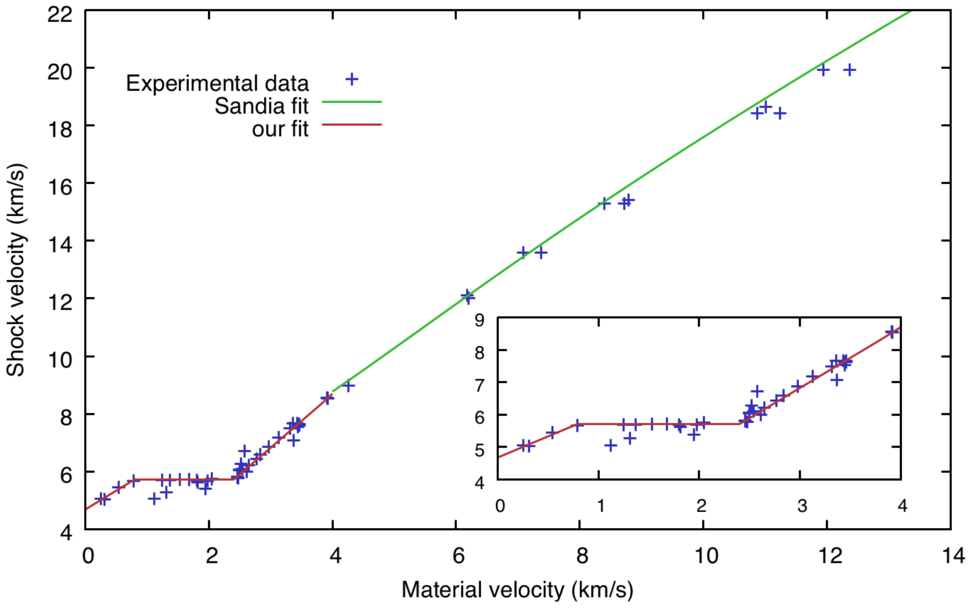

Supplementary Figure 1. Shock – material velocity relation for α-quartz showing experimental data [3–9], the fit done at the Sandia facility by Knudson & Desjarlais (2009) [2], and our interval-defined fit, performed between 0-4 km/s, below the range of validity of the Sandia fit. Inset: zoom of the same plot at low-pressures, to highlight our fit at conditions where the Sandia model cannot be used.

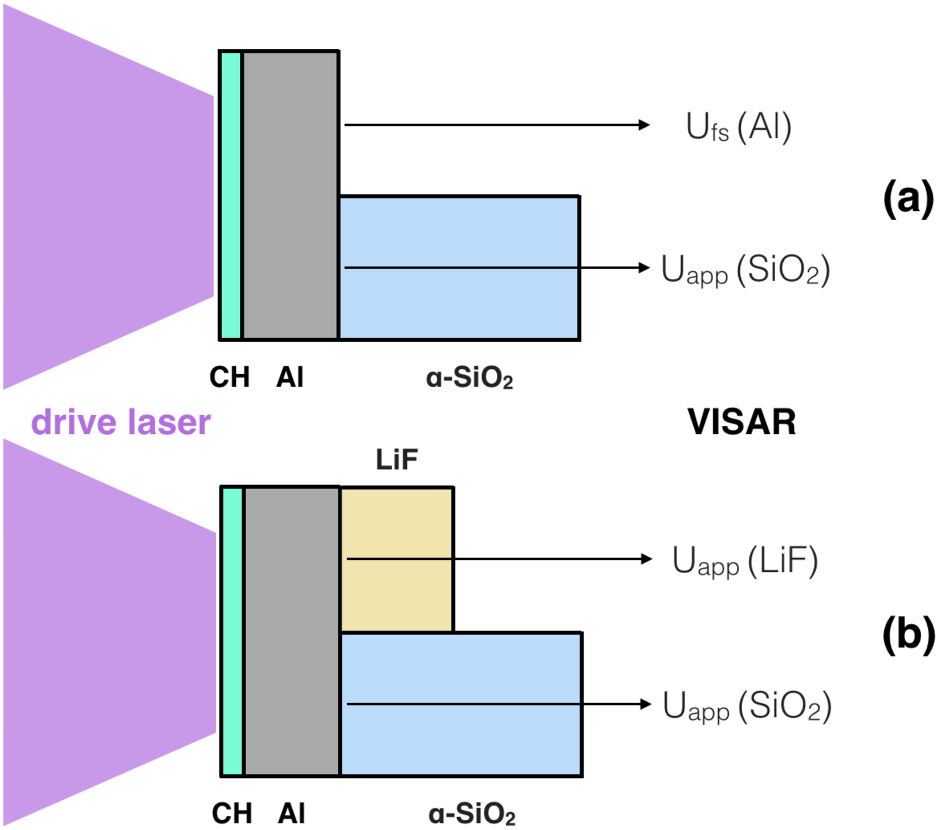

Supplementary Figure 2. Scheme of the target types (a) and (b) for the measurement of the quartz refractive index. The target type (a) allows the simultaneous measure of the free-surface velocity of aluminum and of the apparent velocity of the aluminum-quartz interface (that is, the apparent material velocity in quartz). The target type (b), instead of the free-surface velocity of aluminum, allow the measure of the apparent velocity of the Al-LiF interface (that is, the apparent material velocity in lithium fluoride).

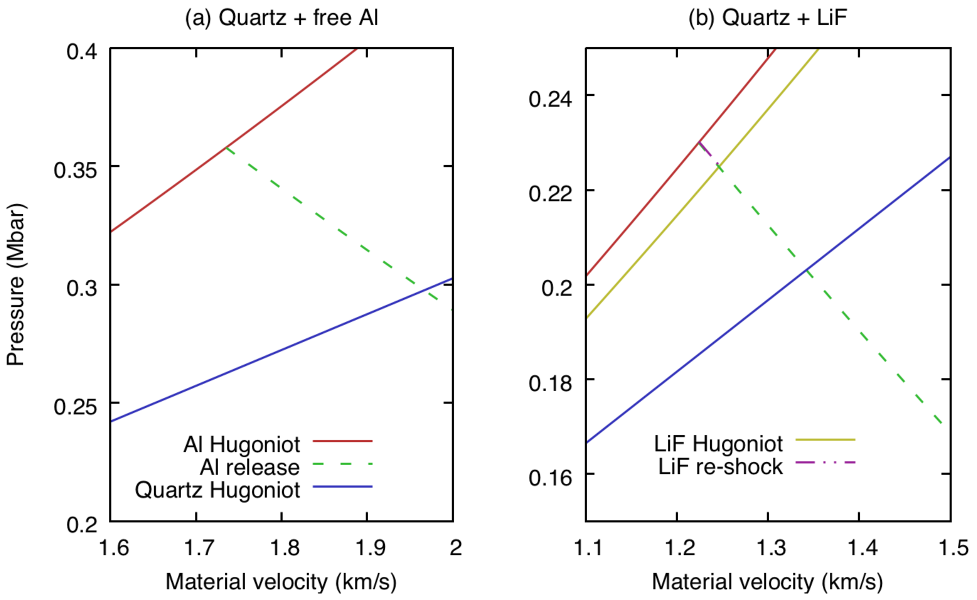

Supplementary Figure 3. Example of impedance mismatch analysis for a shot on a quartz + free-aluminum target (a) and on a quartz + lithium fluoride target (b) for the determination of the shocked quartz state in the context of the measurement of the quartz refractive index.

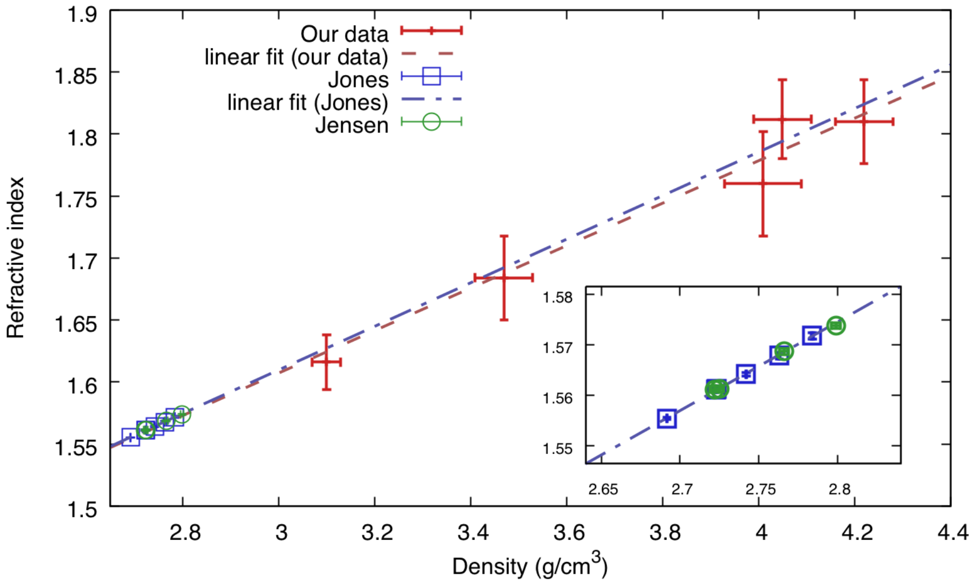

Supplementary Figure 4. Refractive index *vs* density for z-cut α-quartz. Our data (also shown in Supplementary Table I) are at 532 nm. Data from Jones & Gupta (2000) [12] (highlighted on the inset) are at 514.5 nm. Both linear fits are forced to reproduce the known refractive index value at standard conditions.

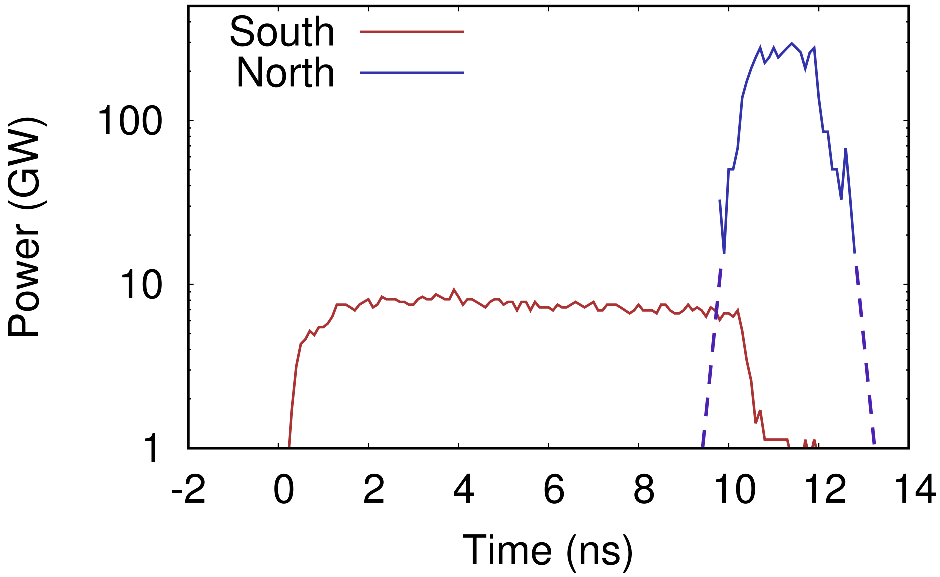

Supplementary Figure 5. Time profile of the drive laser pulses for shot A-40.

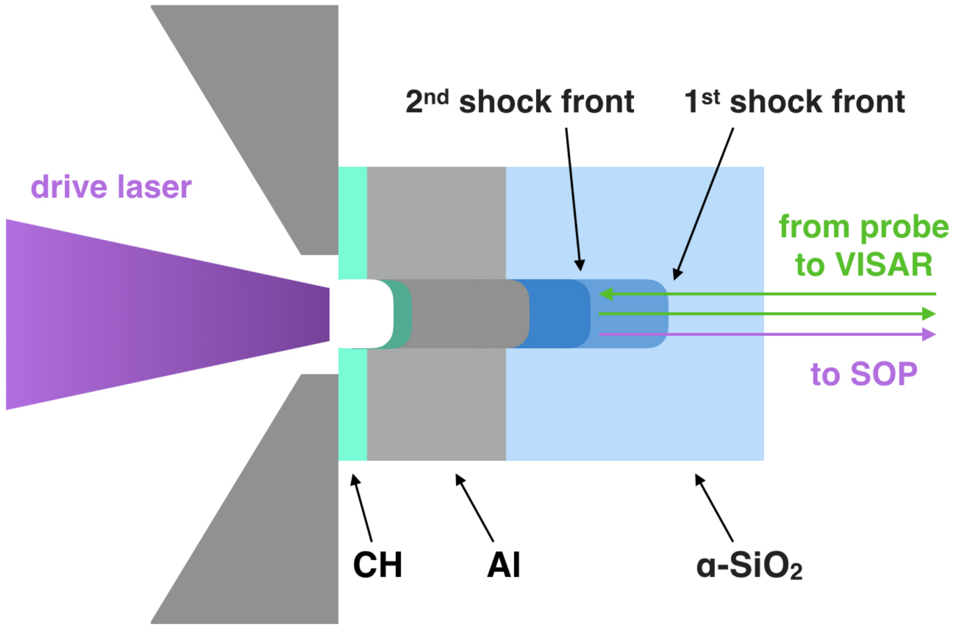

Supplementary Figure 6. Schematic picture of the target during the propagation of the two shock fronts inside the α-quartz sample. The focal spot is 500 μm.

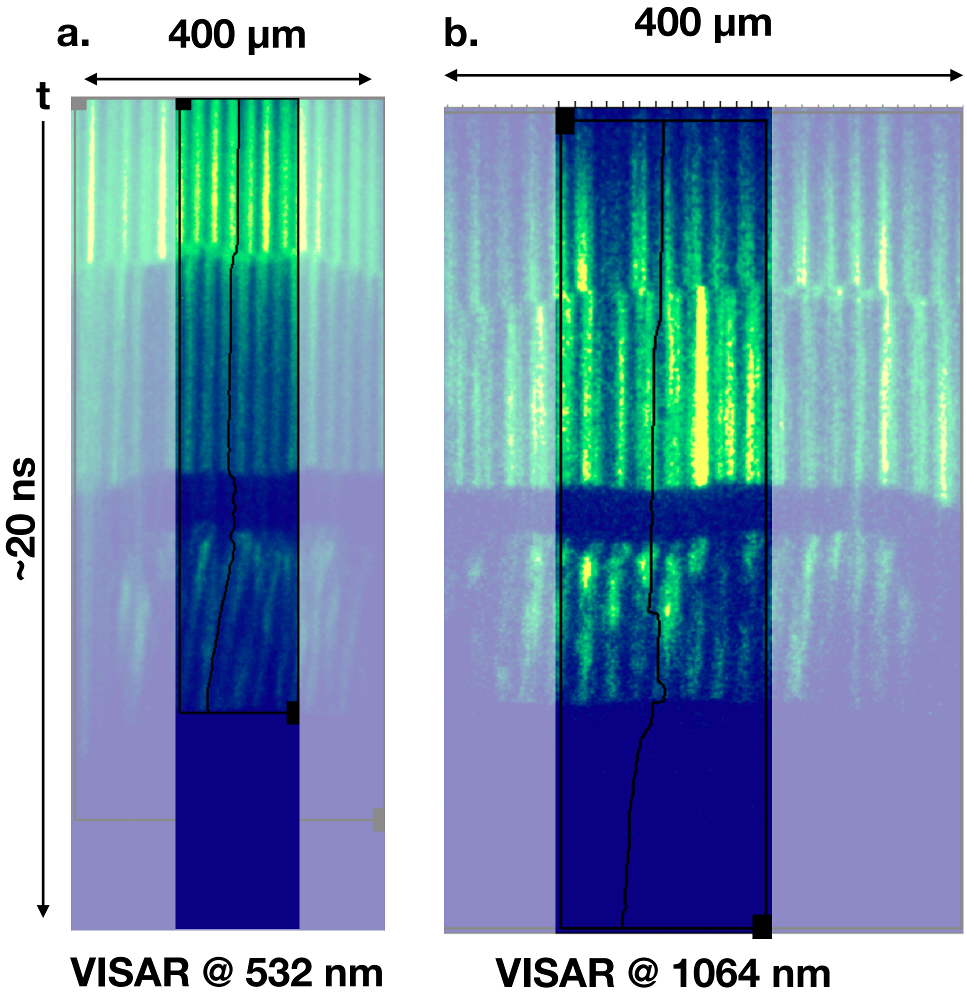

Supplementary Figure 7. Examples of the raw data for the 532 nm (a) and 1064 nm (b) VISAR. Time goes from top to bottom and the time window is about 20 ns. A typical region considered for the analysis is highlighted.

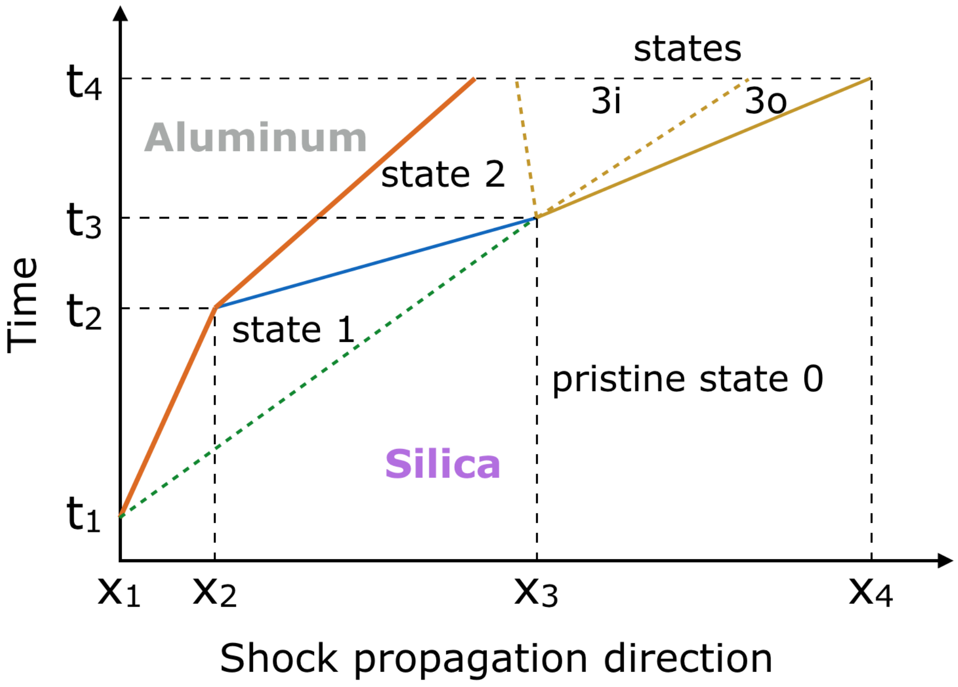

Supplementary Figure 8. Scheme of the propagation of the shocks in the silica sample, in the time-space plane. The bold orange line represents the aluminum/silica interface. Un-shocked silica initially is in the pristine state 0. The first shock front is the dashed green line, behind which silica is loaded to state 1. The second shock front is the solid blue line, behind which silica is loaded to state 2. The two shocks merge at $x = x_3$ and $t = t_3$. The merged shock front is the solid yellow line. An adiabatic release wave starts propagating to the left and the cold front to the right (the two dashed yellow lines). The adiabatic release wave connects state 2 and state 3i; the cold front connects state 3i and state 3o; the merged shock wave loads pristine alpha-quartz (state 0) to state 3o, which lies along the principal Hugoniot of alpha-quartz.

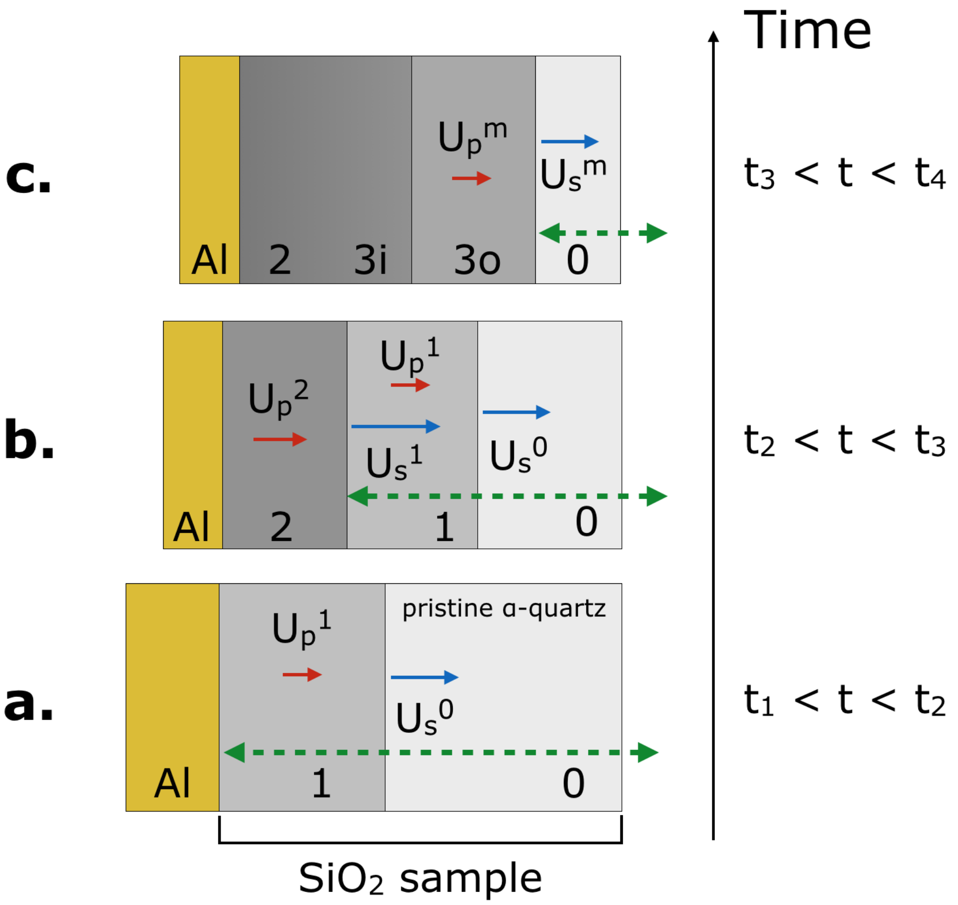

Supplementary Figure 9. Structure of the aluminum and silica layers of the target during the compression (from bottom to top): a. the propagation of the first shock in the sample; b. the propagation of both shocks in the sample; c. the propagation of the merged shock in the sample, as the result of the merging of the two shock fronts. The blue vectors represent the shock velocities; the red vectors represent the fluid velocities. The green dashed arrow shows which interface is probed by the VISARs at each time interval.

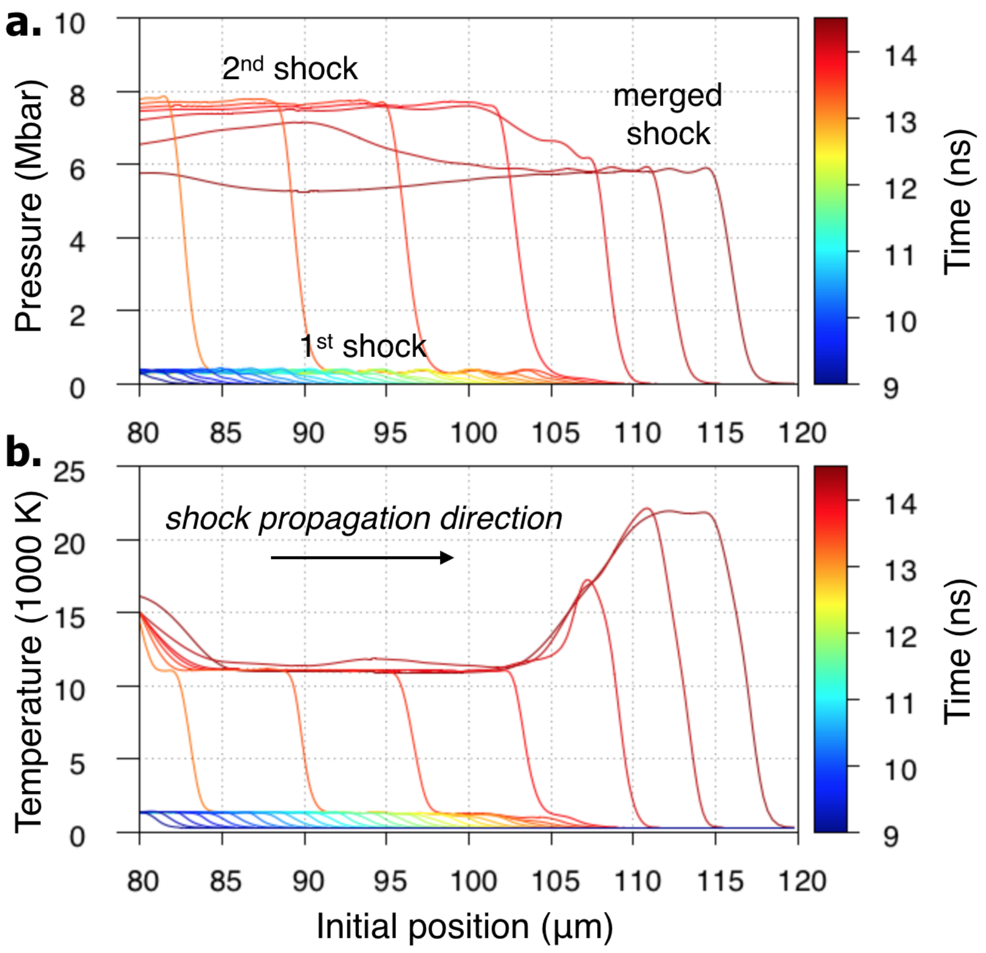

Supplementary Figure 10. Output of a 1-D hydrodynamic simulation with the code MULTI of a double-shock shot on a target composed by 10 μm of polystyrene, 70 μm of aluminum, and 500 μm of alpha-quartz. Temporal profile of the pressure (a) and of the temperature (b) as a function of the position in the target. Only the silica sample is represented. The pressure behind the second shock is slightly higher than that behind the merged shock (on-Hugoniot), while the temperature is significantly lower.

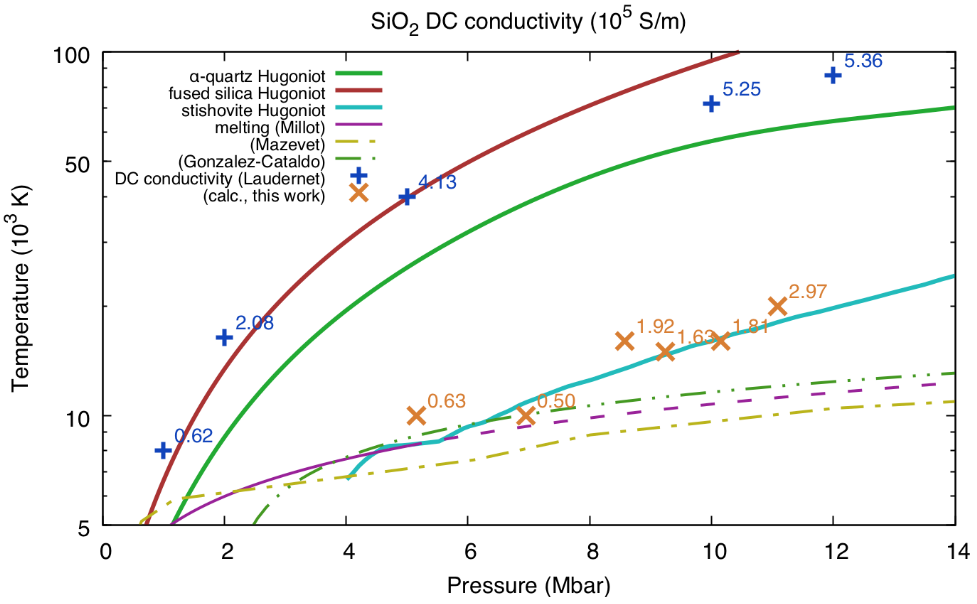

Supplementary Figure 11. Static conductivity of silica according to calculations from Laudernet *et al.* [16] and from our present work. Hugoniot curves of the fused silica [17], α-quartz [18] and stishovite [18], as well as melting line predictions [18–20] are plotted for reference.

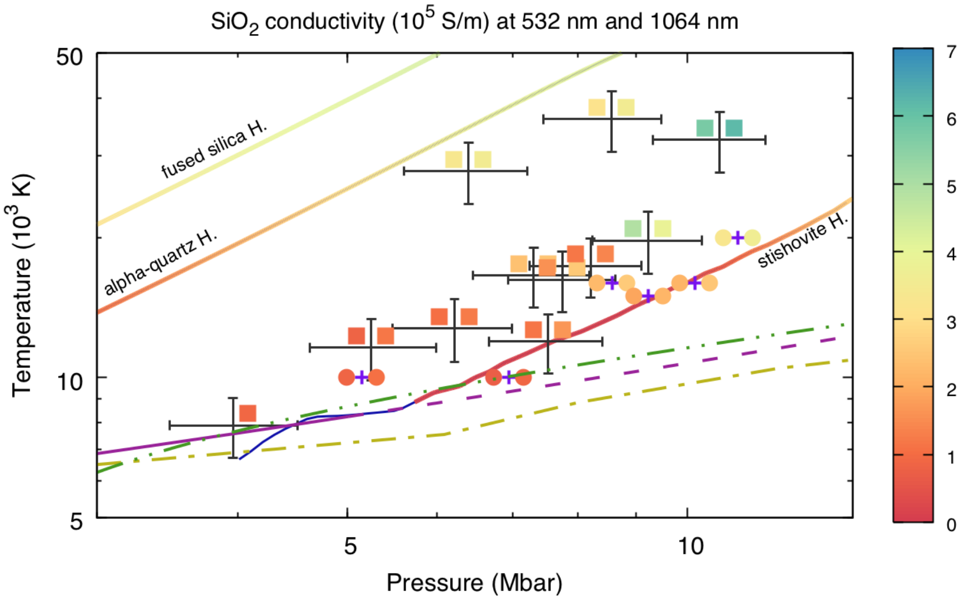

Supplementary Figure 12. Optical conductivities (real part) of warm dense silica. Grey crosses are the conditions experimentally explored via double-shocks; violet crosses are the conditions simulated in this work. Coloured squares (experimental data) and circles (calculations) on the left and right side of the crosses express the conductivity at 1064 nm and 532 nm, respectively, according to the colour scale. Solid curves represent the real part of optical conductivity at 532 nm along the fused siica, alpha-quartz and stishovite Hugoniot estimated with our method using reflectivity values found in literature [17, 18]. The melting curves of silica are the same as in Supplementary Figure 11.

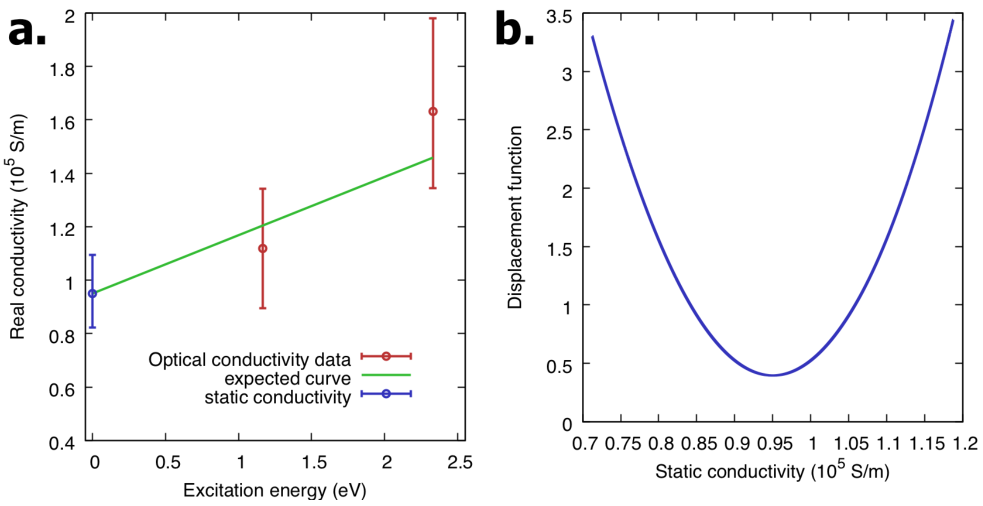

Supplementary Figure 13. Determination of the static conductivity of the double-shocked state in shot D-54. a. Real part of the conductivity as a function of the photon energy. An energy of 1.165 eV (2.331 eV) corresponds to a wavelength of 1064 nm (532 nm). b. Displacement function (introduced in equation 12 against tentative static conductivity values. The minimum of the displacement function is found for a conductivity value of 0.95×10^5 S/m.

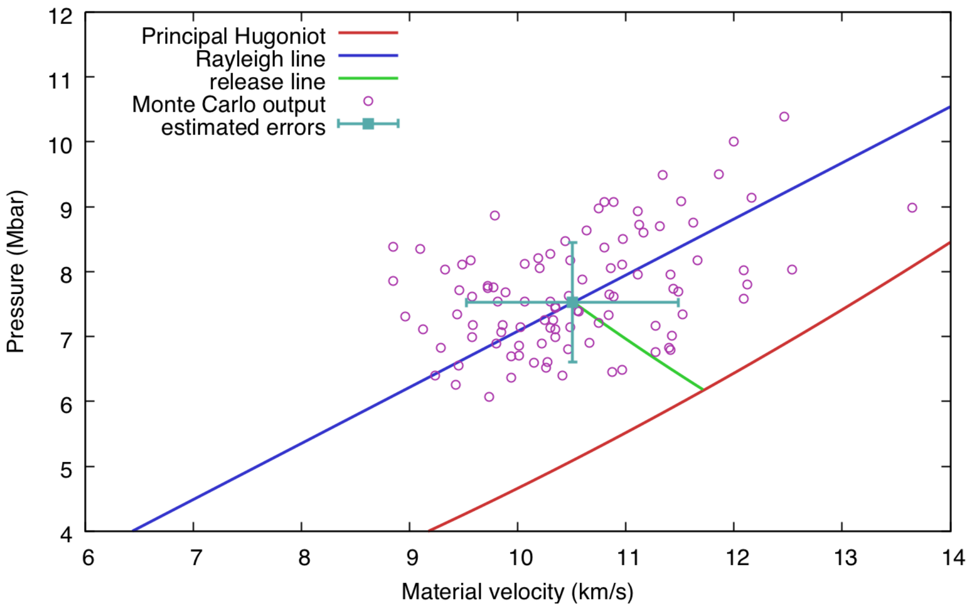

Supplementary Figure 14. Monte-Carlo error estimation of the double-shocked state, determined via self impedance mismatch in the P-U_{p} plane.

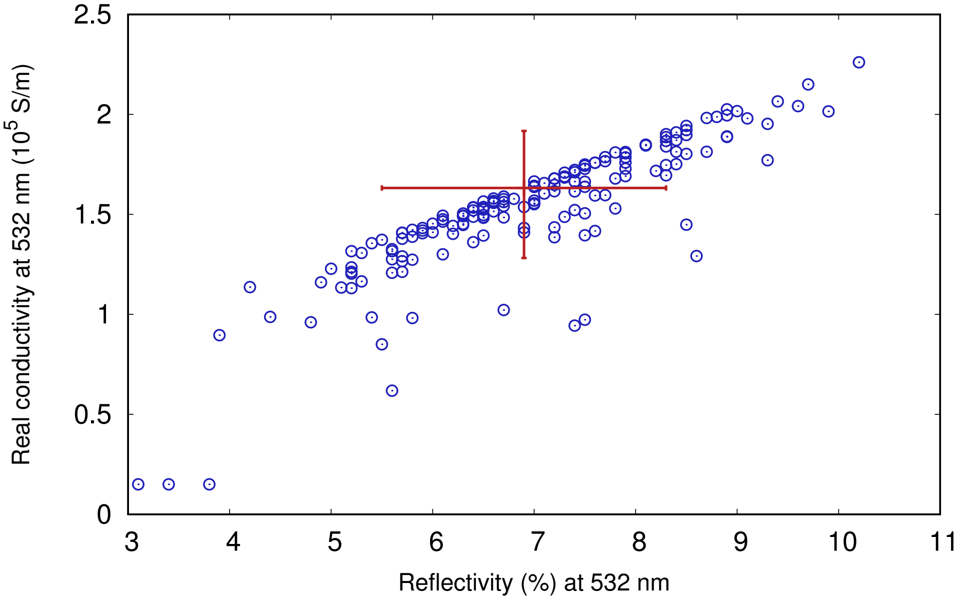

Supplementary Figure 15. Monte-Carlo error estimation of the electrical conductivity at 532 nm. The Monte-Carlo real part of the conductivity at that wavelength is shown as a function of the Monte-Carlo reflectivity value at the same wavelength.

[1] Guarguaglini, M. *et al.* Characterizing equation of state and optical properties of dynamically pre-compressed materials. *Physics of Plasmas* **26**, 042704 (2019).

[2] Knudson, M. D. & Desjarlais, M. P. Shock Compression of Quartz to 1.6 TPa: Redefining a Pressure Standard. *Physical Review Letters* **103**, 225501 (2009).

[3] Adadurov, G., Dremin, A., Pershin, S., Rodionov, V. & Ryabinin, Y. N. Shock compression of quartz. *Zh. Prikl. Mekh. Tekhn. Fiz.* **4**, 81–89 (1962).

[4] L. V. Al'tshuler, G. V. S., R. F. Trunin. Shock compression of periclase and quartz and composition of low earth's mantle. *Izv. Akad. Nauk. SSSR Fiz. Zemli* **10**, 1–6 (1965).

[5] Trunin, R. F., Simakov, G. V., Podurets, B. N., M.A. andMoiseev & Popov, L. Dynamical compressibility of quartz and quartzite at high pressures. *Izv. Akad. Nauk. SSSR Fiz. Zemli* **1**, 13–20 (1971).

[6] Pavlovskii, M. N. Measurements of sound velocity in quartzite, dolomite, anhydrite, sodiumn cloride, paraffin, plexiglas, polyethilene, and fluoroplast-4 under shock compression. *Zh. Prikl. Mekh. Tekhn. Fiz.* **5**, 136–139 (1976).

[7] van Thiel, M. Compendium of shock wave data. *Lawrence Livermore Laboratory Report* **UCRL-50108**, 373–376 (1977).

[8] Marsh, S. P. *LASL Shock Hugoniot Data* (Univ. California Press, 1980).

[9] Trunin, R. Shock compressibility of condensed materials in strong shock waves generated by underground nuclear explosions. *Physics - Uspekhi* **37** (1994).

[10] Boettger, J. C. New model for the shock-induced α-quartz $\rightarrow$ stishovite phase transition in silica. *Journal of Applied Physics* **72**, 5500–5508 (1992).

[11] Ghosh, G. Dispersion-equation coefficients for the refractive index and birefringence of calcite and quartz crystals. *Optics Communications* **163**, 95–102 (1999).

[12] Jones, S. C. & Gupta, Y. M. Refractive index and elastic properties of z-cut quartz shocked to 60 kbar. *Journal of Applied Physics* **88**, 5671–5679 (2000).

[13] Jensen, B. J., Holtkamp, D. B., Rigg, P. A. & Dolan, D. H. Accuracy limits and window corrections for photon Doppler velocimetry. *Journal of Applied Physics* **101**, 013523 (2007).

[14] Knudson, M. D. *et al.* Near-absolute Hugoniot measurements in aluminum to 500 GPa using a magnetically accelerated flyer plate technique. *Journal of Applied Physics* **94**, 4420–4431

(2003).

[15] Rigg, P. A., Knudson, M. D., Scharff, R. J. & Hixson, R. S. Determining the refractive index of shocked [100] lithium fluoride to the limit of transmissibility. *Journal of Applied Physics* **116**, 033515 (2014).

[16] Laudernet, Y., Clérouin, J. & Mazevet, S. Ab initio simulations of the electrical and optical properties of shock-compressed SiO2. *Phys. Rev. B* **70**, 165108 (2004).

[17] Hicks, D. G. *et al.* Dissociation of Liquid Silica at High Pressures and Temperatures. *Physical Review Letters* **97**, 025502 (2006).

[18] Millot, M. *et al.* Shock compression of stishovite and melting of silica at planetary interior conditions. *Science* **347**, 418–420 (2015).

[19] Mazevet, S., Tsuchiya, T., Taniuchi, T., Benuzzi-Mounaix, A. & Guyot, F. Melting and metallization of silica in the cores of gas giants, ice giants, and super Earths. *Physical Review B* **92**, 014105 (2015).

[20] González-Cataldo, F., Davis, S. & Gutiérrez, G. Melting curve of SiO_2 at multimegabar pressures: implications for gas giants and super-Earths. *Scientific Reports* **6**, 26537 (2016).
